# All-boron analogue of planar benzene on an osmium template

**DOI:** 10.1039/d5sc06992k

**Published:** 2025-10-24

**Authors:** Ketaki Kar, Gaurav Joshi, Eluvathingal D. Jemmis, Sundargopal Ghosh

**Affiliations:** a Department of Chemistry, Indian Institute of Technology Madras Chennai 600036 India sghosh@iitm.ac.in; b Inorganic and Physical Chemistry Department, Indian Institute of Science Bangalore 560012 India jemmis@iisc.ac.in

## Abstract

The synthesis of the planar hexaborane B_6_ ring remains a long-standing and elusive ambition in boron chemistry, defying boron’s intrinsic electron deficiency that drives it to favour polyhedral three-dimensional geometries rather than chains and rings. Owing to this domination, earlier attempts to stabilize the planar B_6_ ring in a monometallic template encountered no success. Herein, we report the synthesis and structure of bis-*nido*-[Cp*Os(*η*^6^-B_6_H_11_)] (Cp* = *η*^5^-C_5_Me_5_) (1), the first planar [B_6_H_11_] ring stabilized by the monometallic late transition metal (TM) fragment [Cp*Os]. The fluxionality due to rapid exchange between the bridging hydrogens in 1 led to considerable structural intrigue, resembling the dynamic behaviour seen in *nido*-[B_6_H_10_]. Furthermore, we have successfully isolated the intermediate boron chains, *nido-arachno*-[Cp*Os(*η*^5^-B_5_H_12_) (2) and *nido-arachno*-[Cp*Os(*η*^4^-B_4_H_9_) (3). The B_5_ chain in 2 and the B_4_ chain in 3 are isoelectronic with the pentadienyl radical (C_5_H_7_) and the 1,3-butadiene radical cation (C_4_H_6_)^+^, respectively. Extensive multicenter bonding interactions are demonstrated to stabilize the unique flat ring, as well as the boron chains within monocapped scaffolds.

## Abstract 2

The first planar B_6_ ring stabilized in the coordination sphere of a monometallic complex has been structurally characterized. It exhibits an aromaticity pattern similar to that of a benzene ring stabilized in double-decker sandwich complexes.

## Graphical abstract

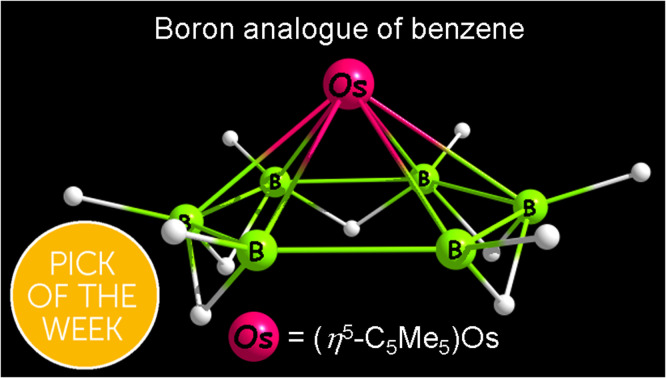

## Introduction

**Chart 1:**
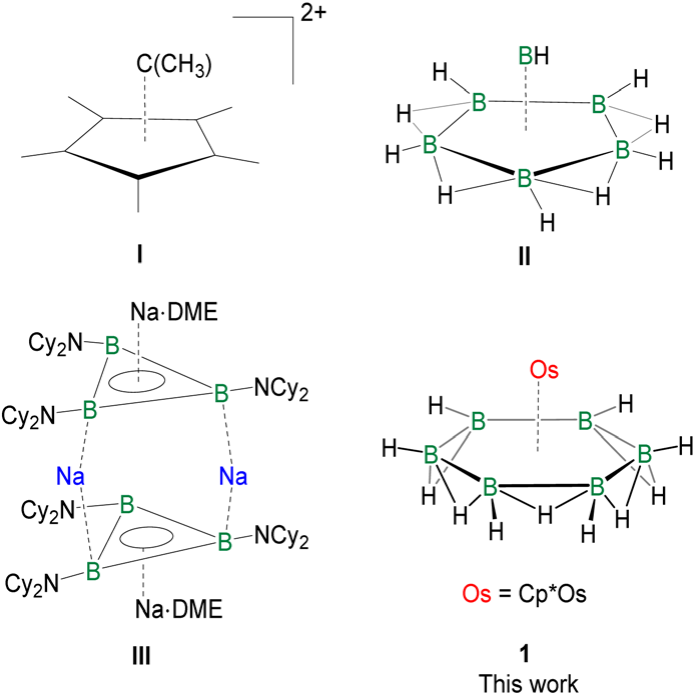
The occurrence of pyramidal geometry in (C_6_(CH_3_)_6_)^2+^ (I), isoelectronic to *nido*-[B_6_H_10_] (II), and planar B_3_ rings in Na_4_[B_3_(NCy_2_)_3_]_2_·2DME (III).

The influence of planar organic aromatic molecules in chemistry is overwhelming; in contrast, the research into the chemistry of aromatic molecules involving neighbouring main group elements is only beginning. Interest in the chemistry of the *cyclo*-P_*n*_ ring, isoelectronic to *cyclo*-(CH)_*n*_, is coming of age.^1–5^ There are now several examples of aromatic systems among heavier elements in the nitrogen group.^6–11^ The boron group presents a different scenario due to electron deficiency.^12–18^ The classical *closo*-polyhedral boranes (B_*n*_H_*n*_)^2−^ follow Wade’s Rule and the Rudolph diagrams exemplify the *nido*- or *arachno*-structures with an increasing number of electrons.^12^ Planar boron rings are rarely in the reckoning. Interestingly, the early synthesis and structural assignment of a dication of hexamethylbenzene, (C_6_(CH_3_)_6_)^2+^ (I), which is isoelectronic to *nido*-[B_6_H_10_] (II), have been a reminder of the lineage to planar rings (Chart 1).^19–22^ Over the past few decades, there have been several reports on planar B_*n*_ rings (*n* = 3–5) stabilized either by main group or transition metal (TM) fragments. For example, B_3_ rings in Na_4_[B_3_(NCy_2_)_3_]_2_·2DME (III) (Chart 1), the B_4_ ring in [(CO)_3_Fe(B_4_H_8_)], the B_5_ ring in [CpFe(B_5_H_10_)], and others.^23–30^ Although numerous theoretical studies have shown the stabilization of the planar B_6_ ring that is isoelectronic with benzene,^31–35^ only a very few experimental reports have demonstrated its stabilization within a triple-decker sandwich-type scaffold using early transition metals.^36–39^ Recently, we have compiled these significant experimental and theoretical advances and proposed a modified Rudolph diagram with an emphasis on planar boron rings.^30^ Despite several significant efforts and occasional breakthroughs in the chemistry of hexagonal planar boron rings, the isolation of the flat parent B_6_ ring, isoelectronic to benzene, in a monometallic template remains elusive.

**Scheme 1:**
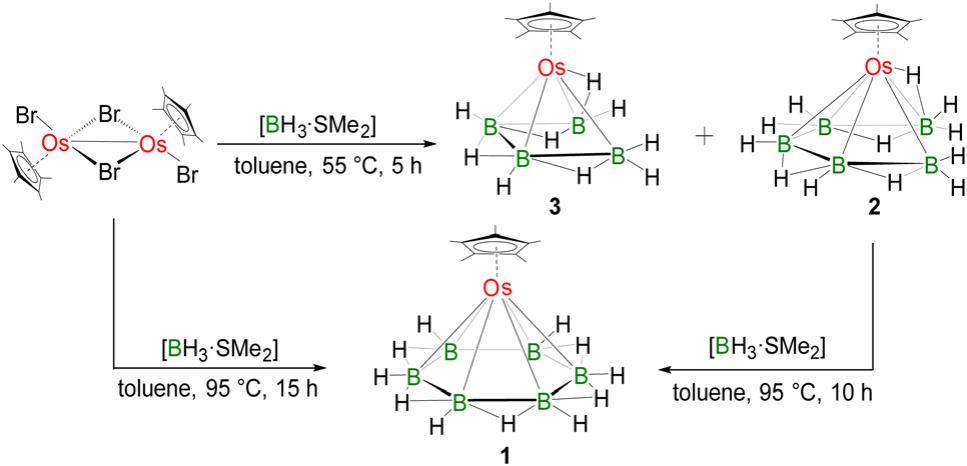
Synthesis of 1 [Cp*Os(*η*^6^-B_6_H_11_)], 2 [Cp*Os(*η*^5^-B_5_H_12_)], and 3 [Cp*Os(*η*^4^-B_4_H_9_)].

Although some reports on planar B_*n*_ (*n* = 4 and 5) rings stabilized in monometallic templates are available, all attempts to isolate a hexagonal planar B_6_ ring in a monometallic template have not met with success. In this article, we report the first planar B_6_ ring stabilized by a monometallic {Cp*Os} unit in bis-*nido*-arrangement, [Cp*Os(*η*^6^-B_6_H_11_)] (1), utilizing an optimized new synthetic strategy involving controlled pyrolysis (Scheme 1). The [B_6_H_11_] ring with five B–H–B bridging hydrogens is isoelectronic to the benzenyl cation radical [C_6_H_6_]^+^. In pursuit of elucidating the intermediates from the monoborane precursor to complex 1 featuring a flat B_6_ ring, we have also isolated key intermediates *nido-arachno*-[Cp*Os(*η*^5^-B_5_H_12_)] (2) and *nido-arachno*-[Cp*Os(*η*^4^-B_4_H_9_)] (3). [B_5_H_12_] in 2 and [B_4_H_9_] in 3 are isoelectronic to pentadienyl radical [C_5_H_7_], and 1,3-butadiene radical cation [C_4_H_6_]^+^, respectively, using less obvious analogies (*vide infra*). Although attempts to isolate the B_2_ and B_3_ intermediates have not been successful, this is the first synthetic attempt to follow reactions starting from B_1_ species to the planar B_6_ ring, isolating and characterizing the intermediate B_4_ and B_5_ chains along the way. Complexes 1, 2, and 3 have been well characterized through various spectroscopic techniques and single-crystal X-ray diffraction analysis.

## Results and discussion

### Synthesis and characterization

The thermolysis reaction of [Cp*OsBr_2_]_2_ with an excess of [BH_3_·SMe_2_] at 95 °C resulted in the formation of [Cp*Os(*η*^6^-B_6_H_11_)] (1) as a colourless solid along with some unidentified air- and moisture-sensitive products. The ^1^H NMR spectrum of 1 shows a peak at *δ* = 1.58 ppm for Cp* that was confirmed by ^13^C{^1^H} NMR. The ^1^H chemical shift at *δ* = −3.56 ppm indicates B–H–B protons. The room-temperature ^11^B{^1^H} NMR spectrum of 1 exhibits a single peak at *δ* = 8.6 ppm. The ^1^H{^11^B} chemical shift at *δ* = 3.56 ppm can be assigned to six terminal B–H protons. The mass spectrum of 1 shows isotopic distribution patterns at *m*/*z* = 402.2301, corresponding to the molecular formula [C_10_H_27_B_6_Os].

**Fig. 1:**
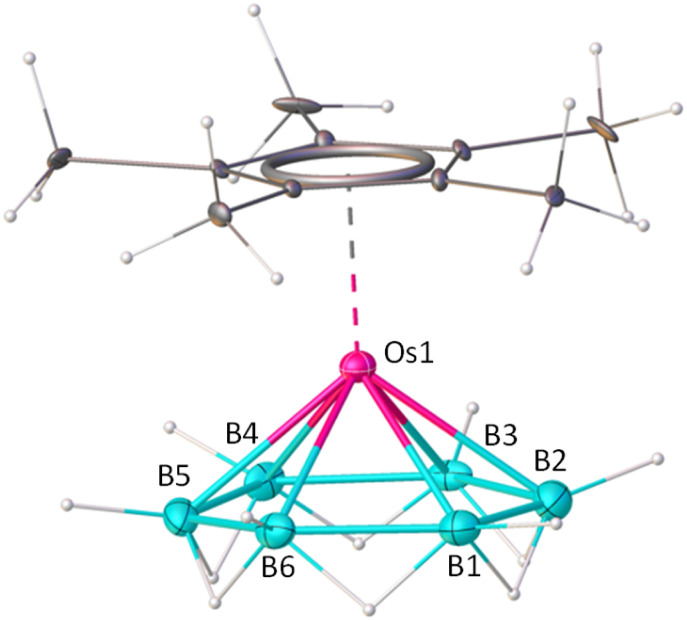
Molecular structure and labelling diagram of 1. Selected bond lengths (Å) and angles (deg.): B1–B2 1.754(17), B2–B3 1.74(3), B3–B4 1.75(2), B4–B5 1.762(14), B5–B6 1.74(2), Os1–B1 2.26(3), Os1–B3 2.20(2), Os1–B5 2.16(3); B4–B5–B6 117(2), B3–B4–B5 123(2). The standard refinement of the X-ray diffraction data for this 18-electron complex 1 indicates the presence of six bridging hydrogens (B–H–B). However, for a neutral 18-electron complex, only 5 bridging hydrogens are expected. Closer analysis of the X-ray diffraction data, supported by theoretical calculations, confirms a structure featuring five bridging hydrogens, as discussed in the text.

**Fig. 2:**
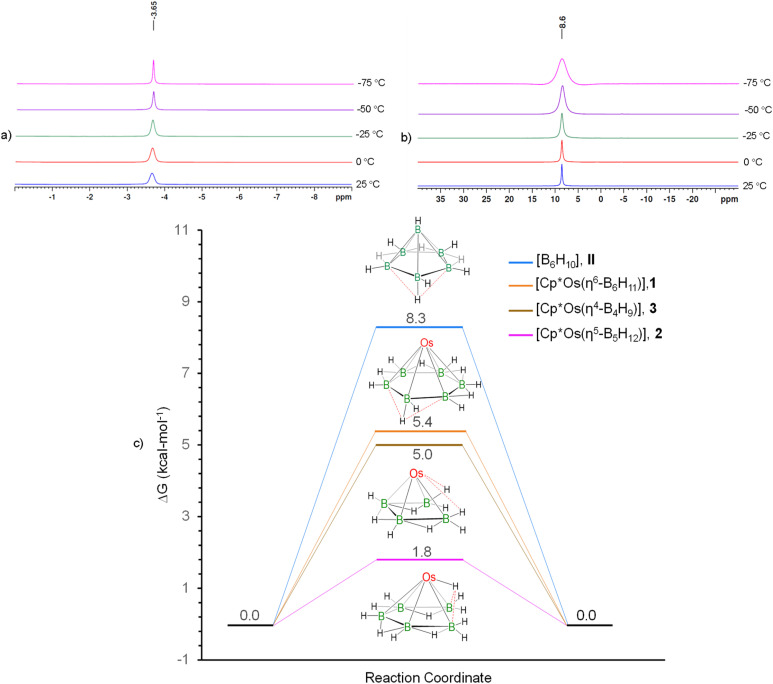
(a) Variable-temperature ^1^H{^11^B} NMR spectra of 1 in the hydride region showing no splitting; (b) variable-temperature ^11^B{^1^H} NMR spectra of 1 showing no splitting except broadening at lower temperatures, (c) free-energy surface for the terminal and bridging hydrogen exchange in [B_6_H_10_] (II) and [Cp*Os(*η*^6^-B_6_H_11_)] (1), the switching of the B–H–Os bridge from one boron atom to another in [Cp*Os(*η*^5^-B_5_H_12_)] (2), and the switching of the agostic interaction in [Cp*Os(*η*^4^-B_4_H_9_)] (3) at the B3LYP-D3/Def2-SVP level of theory with implicit solvation at 298 K. For easy comparison of the variation in the barrier for the hydrogen shifts, the relative energies of the ground states of all compounds are set as zero in the figure.

To corroborate the spectroscopic data, a single-crystal X-ray diffraction analysis was carried out on a suitable crystal of 1. The molecular structure of 1, shown in Fig. 1, shows a planar six-membered B_6_ ring stabilized by a monometallic {Cp*Os} unit. There are six terminal B–H bonds and an additional six hydrogens are positioned to bridge the six B–B bonds forming a [B_6_H_12_] motif with a symmetrical environment. The average Os–B bond distance (2.187 Å) is comparable with the previously reported osmaboranes.^40^ The average B–B bond distance of 1.753 Å in 1 is comparable with the reported triple-decker sandwich complex, *nido-closo-nido*-[(Cp*Re)(*µ-η*^6^:*η*^6^-1,2-B_6_H_4_Cl_2_)(Cp*Re)]^36^ (1.726 Å), but slightly shorter than that of *nido-closo-nido*-[(Cp*Ti)(*µ-η*^6^:*η*^6^-B_6_H_6_)(*µ*-H)_6_(Cp*Ti)]^37^ (1.80 Å). The average ∠B–B–B angle in the six-membered ring is 119.98°, similar to that of a planar hexagon (120°). The molecular formula deduced from the crystal structure, *i.e.*, [Cp*Os(*η*^6^-B_6_H_12_)], leads to an electron count around the metal of 19 (1 × {Cp*Os} = 13, {B_6_H_12_} = 6), suggesting the presence of an odd electron. However, no paramagnetic behaviour was observed in the EPR spectrum of 1. Also, computational studies of the structure [Cp*Os(*η*^6^-B_6_H_12_)], at the B3LYP-D3/Def2-SVP level of theory, with the odd electron, led to a non-planar B_6_ ring (Fig. S29). Furthermore, there is no evidence of the presence of an anion in the unit cell of 1. We examined the X-ray diffraction data further. The terminal B–H and bridging B–H–B protons were identified from difference electron density maps, and their positions were refined using single-crystal X-ray analysis. It was found that all the bridging hydrogen sites are partially occupied with equal occupancy. This partial occupancy indirectly suggests the presence of five bridging hydrogen atoms in 1. Therefore, it is reasonable to assume that the correct formula for 1 is [Cp*Os(*η*^6^-B_6_H_11_)], which results in an 18-electron count around the osmium center (13 electrons from {Cp*Os} and 5 from the {B_6_H_11_}). [B_6_H_11_] is isoelectronic to the benzenyl cation radical [C_6_H_6_]^+^, a 5π ligand. The five additional hydrogens bridge the five out of six B–B bonds and contribute the necessary five electrons. As expected, the non-bridged 2-center-2-electron (2c–2e) B–B bond is computed to be shorter (1.635 Å at the B3LYP-D3/Def2-SVP level) than the bridged 3c–2e B–H–B bond (1.761–1.808 Å, Fig. S30a, and Table S1). The distribution of molecules in the crystal structure is such that the position of the B–B bond without a bridging hydrogen in the crystal structure is random, resulting in diffraction data supporting nearly equal B–B bond distances. The sixth 2c–2e B–B bond is also capable of forming a similar bridged B–H–B 3c–2e bond. Therefore, to check the fluxionality of complex 1, variable temperature ^1^H and ^11^B{^1^H} NMR experiments were performed (Fig. 2a and b), enabling the possibility of slowing down the exchange of five bridged hydrogen atoms. But this experiment revealed no splitting of the single ^11^B peak except broadening at lower temperatures. This broadening may be due to the decrease in spin-lattice relaxation time, a common phenomenon for boron-containing complexes. Probably, a rapid hydrogen exchange among the bridging hydrogens in solution led to the equivalence of five bridging hydrogens. Such a type of rapid exchange of bridging hydrogens has been reported in *nido*-[B_6_H_10_] (II), producing similar ambiguities.^21^ The barrier for the exchange of bridging hydrogens in 1 is calculated to be 5.4 kcal mol^−1^, much lower than the corresponding barrier for II*nido*-[B_6_H_10_] (8.3 kcal mol^−1^) at the same level of theory (Fig. 2c). Therefore, due to the very high fluxionality of the bridging hydrogen atoms, the expected splitting in the variable-temperature ^11^B{^1^H} and ^1^H NMR experiments was not observed, even at very low temperatures. The difficulty in locating the light H atom among a cluster of heavier atoms, together with the possibility of random positioning of the five bridging H-atoms in the B_6_ ring in the crystal structure, leads to the diffraction data that gives six bridging hydrogens instead of five.

### Intermediates

To gain insights into the formation of 1, the reaction of [Cp*OsBr_2_]_2_ with [BH_3_·SMe_2_] was monitored under various reaction conditions. We observed that under milder reaction conditions, we could isolate the lower boron congeners [Cp*Os(*η*^5^-B_5_H_12_)] (2) and [(Cp*Os)(*η*^4^-B_4_H_9_)] (3). Further thermolysis of the mixture of 2 and 3 in the presence of [BH_3_·SMe_2_] at 95 °C for 10 h resulted in the formation of 1. The ^1^H NMR spectrum showed peaks at *δ* = 1.48 ppm (2) and 1.56 ppm (3) for Cp* protons. Peaks in the ^1^H{^11^B} NMR spectrum at *δ* = −3.36 and −3.89 ppm in a 2 : 2 ratio (for 2) and *δ* = −3.79 and −4.16 ppm in a 1 : 2 ratio (for 3) correspond to different B–H–B protons. The upfield ^1^H chemical shifts at *δ* = −12.55 and −15.60 ppm may correspond to terminal Os–H or Os–H–B protons, which were further confirmed by ^1^H–^11^B HSQC as Os–H–B protons (Fig. S14 and S24). The room-temperature ^11^B{^1^H} NMR spectrum of 2 exhibited peaks at *δ* = 2.9, 0.8 and −6.5 ppm in a 2 : 1 : 2 ratio, while 3 exhibited peaks at *δ* = −2.5 and −12.1 ppm in a 2 : 2 ratio. The mass spectra of 2 and 3 demonstrated isotopic distribution patterns at *m*/*z* = 390.2105 and 419.2204, respectively.

**Fig. 3:**
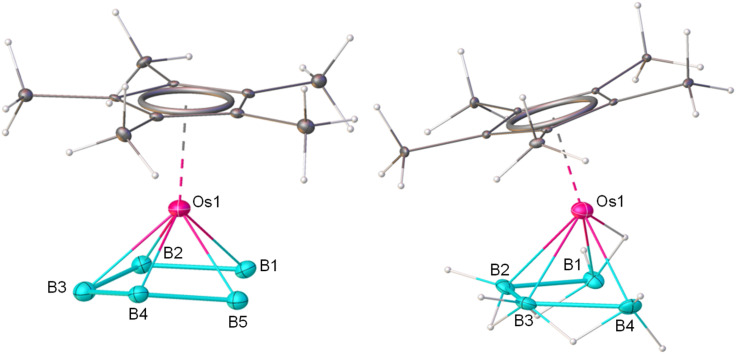
Molecular structures of (2) Cp*Os(*η*^5^-B_5_H_12_) (left) and (3) (Cp*Os)(*η*^4^-B_4_H_9_) (right). Hydrogen positions in 2 could not be located crystallographically due to disorder. They were confirmed by different NMR experiments and computational analysis. Selected bond lengths (Å) and angles (deg.) for 2: Os1–B2 2.02(3), Os1–B3 2.15(3), Os1–B5 2.16(3), Os1–B4 2.23(3), Os1–B1 2.24(3), B1–B2 1.77(4), B3–B4 1.77(4); B2–Os1–B3 57.0(12), B3–Os1–B5 81.9(17), B1–B3 118(3), B4–B2 118(3), B5–B3 107(3). Selected bond lengths (Å) and angles (deg.) for 3: B1–B2 1.846(11), B2–B3 1.826(12), B3–B4 1.851(13), Os1–B1 2.305(7), Os1–B2 2.146(7), Os1–B3 2.150(7), Os1–B4 2.300(7); B3–B1 111.7(6), B2–B4 112.4(5).

The solid-state X-ray structure of 2 confirmed that a B_5_ chain is stabilized in the coordination sphere of the {Cp*Os} unit and this is best described as a *nido-arachno* arrangement (Fig. 3). The average B–B bond distance of 1.822 Å is considerably longer than the average B–B bond distance of 1.713 Å in the triple-decker sandwich complex [(Cp*W)_2_B_5_H_9_].^41^ Unfortunately, due to the presence of two-fold disorder, it was not possible to locate or freely refine any of the 12 hydrogens associated with Os–H–B, B–H–B and terminal B–H. Thus, the hydrogen positions were inferred based on various NMR experiments (Fig. S10, S13 and S14) and computational studies (Fig. S30b). Interestingly, the [B_5_H_12_] chain in 2 is isoelectronic to the pentadienyl radical, [C_5_H_7_], a 5π ligand. This isoelectronic equivalence is most easily demonstrated by assuming [C_5_H_7_] as an anion [C_5_H_7_]^−^ with 6π electrons as in [Cp]^−^. Replacement of five carbon atoms in [C_5_H_7_]^−^ by five isoelectronic [B]^−^ units results in the formula [B_5_H_7_]^6−^. The four B–B sigma bonds can be protonated so that the charge is reduced to −2 in [B_5_H_11_]^2−^. If one of the end [BH_2_] groups (say B1) is also protonated, the resulting [B_5_H_12_]^−^ will have the end boron as an equivalent of [BH_4_]^−^. Thus, there are four π electrons from B2 to B5 and two electrons from one of the B–H bond pairs of B1 to constitute the 6-electron equivalent of [Cp]^−^. The computed structure clearly indicates a stretched B1–H–Os1 interaction, where the B1–H bond is long (1.482 Å at the B3LYP-D3/Def2-SVP level of theory) and the Os1–H distance of 1.657 Å is comparable to the metal–hydride bond distance of 1.661 Å in [Os(PPh_3_)_2_H_2_(*η*^4^-B_4_H_8_)].^29^ The distance between the hydrogen of the B5–H–Os1 3c–2e bond and the terminal B5 atom, *i.e.*, B5–H, is 1.966 Å. If the protonation is assumed to take place at that end (B5), there will be an equivalent structure with B5–H–Os1 3c–2e bond. The barrier for the interconversion between these two equivalent structures is calculated to be only 1.8 kcal mol^−1^ (Fig. 2c). A similar phenomenon was observed with *arachno*-[B_5_H_11_].^22^ Due to this very fast exchange, the expected change in the variable-temperature ^11^B{^1^H} and ^1^H NMR experiments was not observed, even at very low temperatures (Fig. S16 and S17). The crystal structure presents a more complex picture. The structure has a two-fold disorder in the ratio 38 : 62. With the disorder and a very heavy osmium atom present, it is impossible to locate the hydrogen atoms bonded to boron from the difference Fourier map and these hydrogen atoms cannot be fixed geometrically. This explains the unusual differences in B–B bond lengths from the X-ray and computational data (Table S1). Although an analogous C_5_ chain was observed in [Cp*Ru(*η*^5^-C_5_H_7_(CH_3_))]^+^,^42^2 is the first example of boron catenation resulting in the formation of a B_5_ chain in the coordination sphere of a monometallic {Cp*Os} unit.

The solid-state X-ray structure of 3 showed a B_4_ chain stabilized in the coordination sphere of a monometallic {Cp*Os} unit and can be described as *nido-arachno* arrangement (Fig. 3). The average B–B bond distance is 1.841 Å, which is comparable to the average B–B bond distance in [W(IMes)(*η*^4^-BH_2_Mes-BMes-BMes-BH_2_Mes)] (1.82 Å).^43^ This molecule contains nine hydrogens, which are linked as Os–H–B, B–H–B, and terminal B–H bonds. Among these, five are terminal, three bridge the B1–B2, B2–B3 and B3–B4 bonds, and one bridges the Os1–B1 bond (Fig. 3). Though Fehlner and co-workers previously characterized an iridium-stabilized B_4_ chain [Cp*Ir(B_4_H_10_)] (only by NMR experiments),^44^3 is the first example of a structurally characterized osmium-stabilized B_4_ chain. The coordinated [B_4_H_9_] chain in 3 is isoelectronic to the 1,3-butadiene radical cation [C_4_H_6_]^+^, a 3π ligand which, in addition, may donate two electrons through an agostic B–H–Os bond. Replacement of four carbon atoms by four isoelectronic [B]^−^ units results in the formula [B_4_H_6_]^3−^. Protonation of the three B–B sigma bonds results in the neutral [B_4_H_9_]. The three bridging hydrogens provide three electrons. A terminal B–H bond from one of the two end [BH_2_] groups (say B1) of the B_4_-chain provides an additional two electrons through the agostic B1–H–Os1 interaction (B1–H = 1.338 Å, Os1–H = 1.797 Å, Os1–B1 = 2.015 Å, Fig. S33). The other [BH_2_] group (say B4) is similarly capable of this interaction, resulting in a degenerate exchange between the terminal boron atoms with an energy barrier of 5.0 kcal mol^−1^ (Fig. 2c). Due to this rapid exchange of Os–H–B bridging hydrogens, no change was observed in the variable-temperature ^11^B{^1^H} and ^1^H NMR spectra (Fig. S26 and S27), similar to that the results for complexes 1 and 2. While catenation and chain structures serve as the foundation of organic chemistry, homonuclear chains in the chemistry of boron are very limited.^43^ Instead, the formation of hypervalent clusters and cages is the norm. Thus, the π-type complexes featuring B_5_ and B_4_ chain units are very novel.

Our attempts to isolate intermediates with two and three boron atoms (B_2_ and B_3_) were not successful in this reaction pathway. Generally, in most of the metathesis reactions between pentamethylcyclopentadienyl metal polychlorides and borane/borate reagents, such as [BH_3_·THF], [BH_3_·SMe_2_], [LiBH_4_], Li[BH_3_(EPh)], (E = S, Se or Te), *etc.*, the typical by-products are [BH_3_], [BHCl_2_], [BH_2_Cl], *etc.* All these reagents underwent salt-elimination reactions leading to the formation of metal polyborohydride complexes that successively yielded metallaborane by hydrogen elimination.^45,46^ So, in this case, the metathesis reactions between pentamethylcyclopentadienyl metal polybromides and [BH_3_·SMe_2_] may have primarily yielded [BH_3_], [BHBr_2_], [BH_2_Br], *etc.*, along with osmium polyborohydride complexes, which may have undergone hydrogen elimination to form complexes 2, 3, and, subsequently, 1. In addition, these reactions also yielded several air- and moisture-sensitive by-products. Although we have tried to isolate other sensitive by-products by fractional crystallization, we were unsuccessful, mainly due to their poor yields. We could isolate only those compounds that are stable during chromatography work-up. This prevents us from formulating an acceptable reaction mechanism currently.

### Electronic structures and bonding

While the electron requirement of 1 is understood in comparison to ferrocene, these structures, especially 2 and 3, are better understood using the *mno* rule, an extension of Wade’s Rule.^47^ According to this, the electron count required for the stability of a condensed polyhedral structure is given by (*m* + *n* + *o*) skeletal electron pairs, where *m* is the number of polyhedra, *n* is the number of vertices, and *o* is the number of polyhedra connected through a single bridging atom. For *nido* and *arachno* arrangements, two additional variables, *p* and *q*, are added, with values of 1 and 2 electron pairs, respectively. Thus, ferrocene requires 16 (*m* = 2, *n* = 11, *o* = 1, *p* = 2) skeletal electron pairs. As 16 electron pairs are available (30 electrons from ten [CH] units, and 2 electrons from the Fe atom), ferrocene is electronically saturated. Application of the *mno* rule demands 17 electron pairs in 1 (*m* = 2, *n* = 12, *o* = 1 and *p* = 2), and the available number is also 17 (15 electrons from five [CH] units, 12 electrons from the [B_6_H_6_] unit, 5 electrons from five bridging H atoms, and 2 electrons from the Os atom). It is instructive to relate the bonding in 1 to the known triple-decker sandwich *nido-closo-nido*-[(Cp*Re)(*µ-η*^6^:*η*^6^-1,2-B_6_H_4_Cl_2_)(Cp*Re)]. While an *mno* electron count of 25 electron pairs (*m* = 3, *n* = 18, *o* = 2 and *p* = 2) is obtained for a model compound *nido-closo-nido*-[(Cp*Re)(*µ-η*^6^:*η*^6^-B_6_H_6_)(Cp*Re)], the number of available electron pairs is only 22 (15 pairs from two Cp*units, 6 pairs from the [B_6_H_6_] ring and one pair from the Re atoms, together assuming a pseudo-octahedral complex with d^6^ Re atoms). However, the number of electrons required for stability in triple-decker complexes depends on many factors, such as the size of the middle ring, the size of the metal atom, substituents on the rings, the metal–metal bond, the spin multiplicity of the complex, *etc.*^48^ These variables disappear when one of the [Cp*Re] groups is removed, leading to the equivalent of 1.

The osmaborane 2 has a *nido*-[Cp*Os] unit and an *arachno*-[OsB_5_H_12_] unit condensed through Os, so that the *mno* rule gives 17 electron pairs (*m* = 2, *n* = 11, *o* = 1, *p* = 1, *q* = 2). The number of skeletal electron pairs available is also 17 (15 electrons from five [CH] units, 10 electrons from the [B_5_H_5_] unit, 4 electrons from four bridging H atoms, 2 electrons from the two additional H atoms on the terminal B atoms, 1 electron from the additional H atom forming the Os–H–B 3c–2e bridge and 2 electrons from the Os atom). The *mno* count for 3, (Cp*Os)(*η*^4^-B_4_H_9_), is 16 skeletal pairs (*m* = 2, *n* = 10, *o* = 1, *p* = 1, and *q* = 2). Only 15 electron pairs appear to be available (15 electrons from five [CH] units, 8 electrons from the [B_4_H_4_] unit, 3 electrons from three bridging H atoms, 2 electrons from the two additional H atoms on the terminal B atoms and 2 electrons from the Os atom). However, a closer look at the structure indicates that one of the terminal B–H bonds of the *arachno*-[OsB_4_H_9_] unit bends towards the metal, making it an agostic-B–H–Os interaction. This completes the required electronic requirement.

**Fig. 4:**
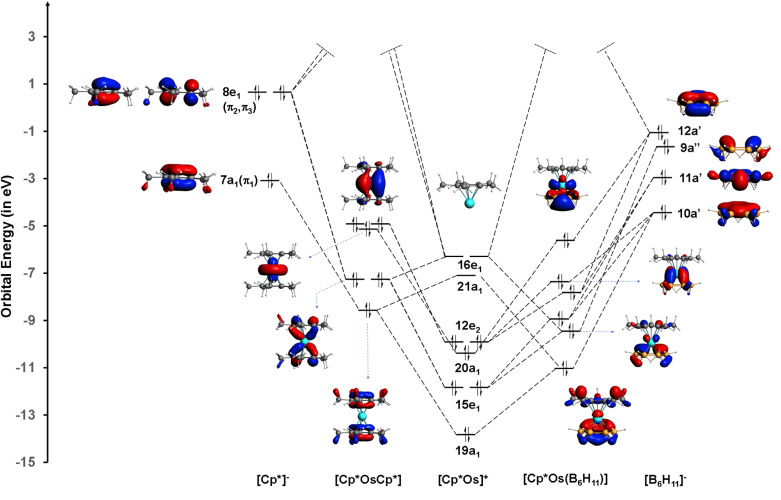
Orbital interaction diagram of the {Cp*Os}^+^ unit with the {Cp*}^−^ and {B_6_H_11_}^−^ ligands in complexes [Cp*OsCp*] and 1 ([Cp*OsB_6_H_11_]), with C_5v_ and C_s_ symmetry, respectively, computed at the B3LYP-D3/TZP level of theory using ADF software.^49,50^ The well-known frontier orbitals of the fragment [Cp*Os] are given in the middle of the diagram. Only the occupied π MOs of Cp* are given on the left. The top four frontier MOs are given for [B_6_H_11_] on the extreme right and there is extensive σ–π mixing in [B_6_H_11_]. C_5v_ point group nomenclature is used for {Cp*}^−^ and {CpOs}^+^, and C_s_ nomenclature for {B_6_H_11_}^−^. In 1, the σ–π mixing makes the distinction between σ and π orbitals difficult, unlike in [Cp*OsCp*].

While this qualitative electron counting brings equivalence between the electronic structure of permethylferrocene and that of [Cp*Os(*η*^6^-B_6_H_11_)] 1, there are many differences in the details. The high stability of the C–C σ bond in Cp* and the high symmetry keep a large difference in the π and σ molecular orbitals in Cp*. In contrast, the five B–B bridging hydrogens in [B_6_H_11_] force the terminal hydrogens onto the opposite side of the B_6_ ring. This leads to σ–π mixing and the distinction between π and σ MOs vanishes. The consequences are seen in Fig. 4, where fragment interaction diagrams for the formation of permethylosmocene and structure 1 are compared. While it is still possible to trace the MOs with a dominant contribution of π MOs of Cp* in permethylosmocene, it is not easily possible to distinguish between orbitals with predominantly σ or π interactions in structure 1, [Cp*Os(*η*^6^-B_6_H_11_)].

**Fig. 5:**
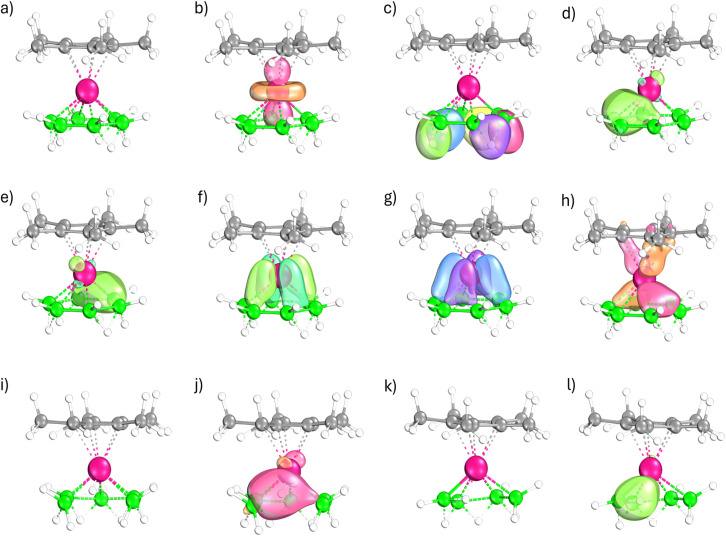
Selected localized orbitals of 1 (a) computed using IBOview; (b) a 1c–2e lone pair on the osmium atom; (c) five 3c–2e bond orbitals; and (d–h) five multicenter osmium–boron bond orbitals. Complexes 2 (i) and 3 (k) display similar localized orbitals, with the exception that in each case one of the five osmium–boron multicenter orbitals include a contribution from a terminal hydrogen atom (j and l, respectively). For clarity, the 2c–2e bond orbitals and three metal–Cp* multicenter orbitals are omitted (see text).

The bonding of 1, 2, and 3 is further examined using Intrinsic Bond Orbitals (IBOs).^51,52^ These are obtained from Intrinsic Atomic Orbitals (IAOs), a minimal set of atomic orbitals on atoms in molecules, polarised to depict the molecular environment. The IBOs derived from IAOs represent the chemical bonding, even in molecules that are difficult to understand. These localized bond orbitals are generated using IBOview and represented for selected bond orbitals of 1, 2 and 3 (Fig. 5). The localization identifies several obvious two-center, two-electron bond orbitals and multicenter bond orbitals (Fig. S31–33). Notably, the metal lone pair (Fig. 5b), the three-center, two-electron orbitals corresponding to the bridging of B–B bonds with hydrogen atoms (Fig. 5c), and three multicenter orbitals connecting the Cp* ring to the metal, remain consistent across all three complexes (Fig. S31–33). The most intriguing variation is the nature of the osmium–boron multicenter bond orbitals as we go from the B_6_ ring in 1 to the B_5_ and B_4_ chains in 2 (Fig. 5i) and 3 (Fig. 5k), respectively. There are five multicenter bond orbitals involving the osmium and boron atoms (Fig. 5d–h for 1, and Fig. S32 and S33 for 2 and 3, respectively). In 2 and 3, one of the multicenter bond orbitals has contributions from the osmium atom and terminal boron atom, and the hydrogen atom bridging them (Fig. 5j and l). In 2, the terminal boron atom is bonded to four hydrogen atoms; two of these are terminal hydrogen atoms, one forms a bridging bond with another boron atom, and one bridges the osmium atom and boron atom. This bonding environment classifies it as a [BH_4_]^−^ fragment. Notably, the B–H bond that interacts with the osmium atom is considerably lengthened, and the osmium atom’s interaction with this terminal boron atom is relatively weak (with B–H, Os–H and Os–B bond distances of 1.482 Å, 1.657 Å, and 2.324 Å, and corresponding bond orders of 0.36, 0.35 and 0.32, respectively). This weakened bond facilitates an easier transfer of hydrogen to another terminal boron atom (Fig. 3). In contrast, in 3, the terminal boron atom is attached to three hydrogen atoms: a single terminal hydrogen atom, one hydrogen atom in a bridging bond with another boron atom, and one in a bridging bond between the osmium and boron, thus assigned as a [BH_3_] fragment. Here, the interacting B–H bond shows only a minor elongation, and the osmium atom has a more pronounced interaction with both the boron atom and the hydrogen atom (with bond distances for B–H, Os–H, and Os–B of 1.339 Å, 1.797 Å, and 2.015 Å, respectively, and bond orders of 0.58, 0.26, and 0.73, respectively). This arrangement characterizes the interaction of a [BH_3_] fragment with an intact B–H bond as an agostic interaction, as described before.

### Aromaticity comparison

We have further compared the aromaticity of the stabilized planar B_6_ ring in the monometallic template (1) with that of the benzene ring in bis(benzene)chromium, [(C_6_H_6_)Cr(C_6_H_6_)]^53^ (Table 1) using NICS (Nucleus Independent Chemical Shift) values. While the absolute NICS values as indicators of aromaticity fail in metal complexes, a comparison can be made between a series of related complexes.^54^ NICS values follow similar trends for 1 and bis(benzene)chromium. The values remain negative at the center and at positions 1 Å above (towards the metal) and below (opposite to the metal) the plane (see the image in Table 1). The negative values away from the metal suggest that 1 is aromatic. Therefore, the planar B_6_ ring follows a similar aromaticity pattern of the benzene ring stabilized in double-decker sandwich complexes. The orbital interaction diagram (Fig. 4, above) shows that, despite the σ–π mixing arising from bridging hydrogens in [B_6_H_11_], the frontier MOs of [Cp*OsB_6_H_11_] (1) have a significant resemblance to those in [Cp*OsCp*],^55^ further highlighting that the aromaticity in 1 should be comparable to [Cp*OsCp*] (Table 1).

**Table 1:** Comparison of NICS values for [Cp*Os(*η*^6^-B_6_H_11_)] (1) and bis(benzene)chromium [(C_6_H_6_)Cr(C_6_H_6_)] and [Cp*OsCp*], computed at the B3LYP-D3/Def2-SVP level of theory. Negative NICS values imply aromaticity. In double-decker sandwich complexes, NICS(1.0) and NICS(−1.0) are measurements towards and away from the metal, respectively; see drawing on the left

	Complexes	NICS_iso_ (0.0)	NICS_zz_ (0.0)	NICS_iso_ (1.0)	NICS_zz_ (1.0)	NICS_iso_ (−1.0)	NICS_zz_ (−1.0)
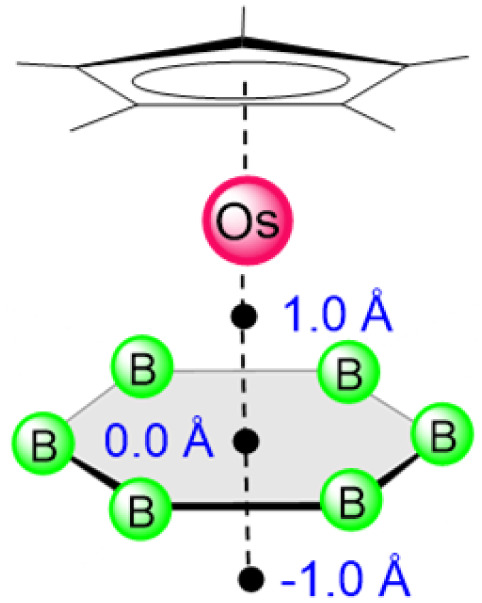	[Cp*Os(*η*^6^-B_6_H_11_)], 1	−37.6	−55.4	−49.5	−166.7	−11.4	−22.1
	[(C_6_H_6_)Cr(C_6_H_6_)]^53^	−48.9	−58.3	−180.0	−229.7	−19.8	−34.0
	[Cp*OsCp*]^55^	−25.9	−30.3	−73.2	−88.2	−12.9	−29.0

## Conclusions

The syntheses of mixed metallocene, [Cp*Os(*η*^6^-B_6_H_11_)] (1), *nido-arachno*-[Cp*Os(*η*^5^-B_5_H_12_)] (2) and *nido-arachno*-[Cp*Os(*η*^4^-B_4_H_9_)] (3) from a B_1_ precursor [BH_3_·SMe_2_] presents novel possibilities in boron chemistry. In complex 1, five bridging hydrogen atoms contribute to an 18-electron-count configuration. In complex 2, four bridging hydrogen atoms, along with an additional hydrogen atom bridging the Os–B bond, achieve the same 18-electron count. In complex 3, although only three bridging hydrogen atoms are present, a B–H bond donates two electrons to the osmium atom in a manner similar to agostic sigma-complex formation, completing the 18-electron configuration. These complexes also follow the *mno* rule. These novel ways in which [B_6_H_11_], [B_5_H_12_] and [B_4_H_9_] provide five electrons lead to dynamic degenerate rearrangements involving H-migrations that cannot be frozen, even at low temperatures. Detailed analysis of aromaticity using NICS values shows that [Cp*Os(*η*^6^-B_6_H_11_)] is nearly as aromatic as bis(benzene)chromium, [(C_6_H_6_)Cr(C_6_H_6_)]. These findings emphasize the intricate bonding environments in all the complexes, with multicenter interactions playing a pivotal role in stabilizing the structures and opening up new approaches in organometallic chemistry without carbon.

## Author contributions

K. K. has executed the experimental synthesis, characterisation and analysed the data. G. J. has carried out theoretical calculations. All authors have contributed to the preparation of the manuscript. S. G. and E. D. J. have supervised the experimental and theoretical studies.

## Conflicts of interest

There are no conflicts to declare.

## Supplementary Material

SC-017-D5SC06992K-s001

SC-017-D5SC06992K-s002

SC-017-D5SC06992K-s003

## Data Availability

The data that support the findings of this study are available in the supplementary information (SI) of this article. Supplementary information: experimental procedures and characterization of all species and reaction products. See DOI: https://doi.org/10.1039/d5sc06992k. CCDC 2438685 (1), 2438789 (3) and 2438887 (2) contain the supplementary crystallographic data for this paper.^56*a*–*c*^
