# Supplementary material for: All-boron analogue of planar benzene on an osmium template
Source: Chem Sci. 2025 Oct 24;17(1):196–204. doi: 10.1039/d5sc06992k (PMC12606463; doi:10.1039/d5sc06992k)
Supplement: SC-017-D5SC06992K-s002 [file SC-017-D5SC06992K-s002.pdf]

Supplementary Information (SI) for Chemical Science.
This journal is © The Royal Society of Chemistry 2025

SUPPORTING INFORMATION

All-Boron Analogue of Planar Benzene on an Osmium Template

Ketaki Kar,[a] Gaurav Joshi,[b] Eluvathingal D. Jemmis,[b] and Sundargopal Ghosh*[a]*

[a]Department of Chemistry, Indian Institute of Technology Madras, Chennai 600036, India. E-mail: sghosh@iitm.ac.in

[b]Inorganic and Physical Chemistry Department, Indian Institute of Science, Bangalore 560012, India. E-mail: jemmis@iisc.ac.in

Table of contents

I Experimental Details

I.1 Spectroscopic Details

Figure S1	ESI-MS spectrum of **1** in CH_2Cl_2	S6
Figure S2	^1H NMR spectrum of **1** in C_6D_6	S6
Figure S3	$^{11}B\{^1H\}$ NMR spectrum of **1** in C_6D_6	S7
Figure S4	$^1H\{^{11}B\}$ NMR spectrum of **1** in C_6D_6	S7
Figure S5	Stacked ^1H (blue) and $^1H\{^{11}B\}$ (red) NMR spectra of **1** in C_6D_6	S8
Figure S6	$^{13}C\{^1H\}$ NMR spectrum of **1** in C_6D_6	S8
Figure S7	Variable temperature ^1H NMR spectra of **1** in d_8-toluene	S9
Figure S8	IR spectrum of **1** in CH_2Cl_2	S9
Figure S9	ESI-MS spectrum of **2** in CH_2Cl_2	S10
Figure S10	^1H NMR spectrum of **2** in C_6D_6	S10
Figure S11	$^{11}B\{^1H\}$ NMR spectrum of **2** in C_6D_6	S11
Figure S12	$^1H\{^{11}B\ \}$ NMR spectrum of **2** in C_6D_6	S11
Figure S13	Stacked ^1H (blue) and $^1H\{^{11}B\}$ (red) NMR spectra of **2** in C_6D_6	S12
Figure S14	^1H-^{11}B HSQC NMR spectrum of **2** in C_6D_6	S12
Figure S15	$^{13}C\{^1H\}$ NMR spectrum of **2** in C_6D_6	S13
Figure S16	Variable temperature ^1H NMR spectra of **2** in d_8-toluene	S13
Figure S17	Variable temperature $^{11}B\{^1H\}$ NMR spectra of **2** in d_8-toluene	S14
Figure S18	IR spectrum of **2** in CH_2Cl_2	S14
Figure S19	ESI-MS spectrum of **3** in CH_2Cl_2	S15
Figure S20	^1H NMR spectrum of **3** in C_6D_6	S15
Figure S21	$^{11}B\{^1H\}$ NMR spectrum of **3** in C_6D_6	S16
Figure S22	$^1H\ \{^{11}B\ \}$ NMR spectrum of **3** in C_6D_6	S16
Figure S23	Stacked ^1H (blue) and $^1H\{^{11}B\}$ (red) NMR spectra of **3** in C_6D_6	S17
Figure S24	^1H-^{11}B HSQC NMR spectrum of **3** in C_6D_6	S17
Figure S25	$^{13}C\{^1H\}$ NMR spectrum of **3** in C_6D_6	S18
Figure S26	Variable temperature ^1H NMR spectra of **3** in d_8-toluene	S18
Figure S27	Variable temperature $^{11}B\{^1H\}$ NMR spectra of **3** in d_8-toluene	S19

Figure S28 IR spectrum of **3** in CH_2Cl_2 S19

I.2 X-ray Details S20

II Computational Details S20

Figure S29 Optimized geometry of [Cp*Os(η^6-B_6H_{12})] S21

Figure S30 Optimized geometry of a) [Cp*Os(η^6-B_6H_{11})], b) [Cp*Os(η^5-B_5H_{12})], and c) [Cp*Os(η^4-B_4H_9)] S21

Table S1. Bond parameters of complexes **1**-**3** S22

Figure S31 Selected localized orbitals of **1** S23

Figure S32 Selected localized orbitals of **2** S24

Figure S33 Selected localized orbitals of **3** S25

Figure S34 Selected π molecular orbitals of [(C_6H_6)Cr(C_6H_6) and [Cp*OsB_6H_{11}] S26

III References S26

I Experimental Details

General Procedures and Instrumentation

All the manipulations were conducted under an Ar atmosphere using standard Schlenk line techniques and a glove box. Solvents were distilled under an Ar atmosphere before use. [BH_3·SMe_2] was used as received (Sigma Aldrich). [Cp*$OsBr_2$]$_2$[1] was synthesized according to the literature method. Thin-layer chromatography was carried out on 250-μm diameter aluminum-supported silica gel TLC plates (MERCK TLC Plates) to separate the reaction mixtures. NMR spectra were recorded by using 500 MHz Bruker FT-NMR spectrometer. The residual solvent protons were used as reference (Benzene-d_6, δ = 7.16 ppm; Toluene-d^8, δ = 2.08, 6.97, 7.01, and 7.09 ppm). The inverse-gated decoupling (zgig) and power-gated (zgpr) pulse sequences, respectively, were used to get the $^{11}B\{^1H\}$ and $^1H\{^{11}B\}$ spectra. All pulse sequences are available in commercial Bruker spectrometer. Variable temperature ^1H decoupled ^{11}B NMR spectra were processed with a backward linear prediction algorithm to remove the broad ^{11}B background signal of the NMR probe and NMR tube.[2,3] The mass spectra were recorded on a Bruker MicroTOF-II Qtof instrument for **1** and Waters Xevo G3 Qtof instrument for **2** and **3**. The IR spectra were recorded on a JASCO 400 FT-IR spectrometer.

Synthesis of [Cp*Os(η^6-B_6H_{11})], 1: Under an Ar atmosphere, in a flame-dried Schlenk flask, [Cp*$OsBr_2$]$_2$ (0.7 g, 0.714 mmol) was suspended in toluene (90 mL) and cooled to -78°C. [BH_3·SMe_2] (8.5 mL) was added and the resultant mixture was slowly warmed to room temperature and kept for thermolysis at 95 °C for 15 h. The solvent was then removed under vacuum, and the residue was dissolved in a hexane/CH_2Cl_2 mixture (90:10) and passed through Celite. The solvent was again evaporated under vacuum, and the residue was purified using silica-gel TLC plates by eluting with hexane/CH_2Cl_2 (90:10) mixture that yielded colourless solid **1** (0.147 g, 25%) complex.

1: MS (ESI$^+$): *m/z* calculated for [$C_{10}H_{26}B_6Os$ + H]$^+$: 402.2308, found: 402.2301; ^1H NMR (500 MHz, C_6D_6, 22 °C): δ = 3.60 (br, 6H, B-H_t), 1.58 (s, 15H, 1×Cp*), −3.56 (br, 5H, B-*H*-B); $^{11}B\{^1H\}$ NMR (160 MHz, C_6D_6, 22 °C): δ (ppm) = 8.6 (br, 6B); $^1H\{^{11}B\}$ NMR (500 MHz, C_6D_6, 22 °C): δ (ppm) = 3.56 (br, 6H, B-H_t), 1.58 (s, 15H, 1×Cp*), −3.56 (s, 5H, B-*H*-B); $^{13}C\{^1H\}$ NMR (125 MHz, C_6D_6, 22 °C): δ (ppm) = 96.0 (C_5Me$_5$), 9.0 (C$_5$*Me*$_5$); IR (dichloromethane, cm^{-1}): $\bar{\upsilon}$ = 2415 (B-H_t).

Synthesis of [Cp*Os(η^5-B_5H_{12})], 2 and **[Cp*Os(η^4-B_4H_9)], 3**: Under an Ar atmosphere, in a moisture-free Schlenk flask, [Cp*$OsBr_2$]$_2$ (0.7 g, 0.714 mmol) was suspended in 90 mL toluene and cooled to -78°C. [BH_3·SMe_2] (8.5 mL) was added to the reaction mixture, and the resultant mixture was slowly warmed to room temperature, kept for thermolysis at 55 °C for 5 h. The

solvent was then removed under vacuum, and the residue was dissolved in hexane and passed via celite. The solvent was again evaporated under vacuum, and the residue was purified using silica-gel TLC plates by eluting with hexane, which yielded colourless solid **2** (0.170 g, 30%) and **3** (0.227 g, 40%) complexes.

2: MS (ESI$^+$): *m/z* calculated for $[C_{10}H_{27}B_5Os - H]^-$: 390.2123, found: 390.2105; ^{1}H NMR (500 MHz, C_6D_6, 22 °C): δ = 3.45 (br, 2H, B-H$_t$), 3.01 (br, 3H, B-H$_t$), 2.58 (br, 2H, B-H$_t$); 1.48 (s, 15H, 1×Cp*), −3.36 (br, 2H, B-*H*-B), −3.83 (br, 2H, B-*H*-B), −12.55 (br, 1H, Os-*H*-B); ^{11}B{^{1}H} NMR (160 MHz, C_6D_6, 22 °C): *δ* (ppm) = 2.9 (br, 2B), 0.8 (br, 1B), −6.5 (br, 2B); ^{1}H{^{11}B} NMR (500 MHz, C_6D_6, 22 °C): *δ* (ppm)= 3.57 (s, B-H$_t$), 3.01 (s, B-H$_t$), 2.55 (s, B-H$_t$); 1.48 (s, 15H, 1×Cp*), −3.36 (s, 2H, B-*H*-B), −3.89 (s, 2H, B-*H*-B), −12.55 (s, 1H, Os-*H*-B); ^{13}C{^{1}H} NMR (125 MHz, C_6D_6, 22 °C): *δ* (ppm) = 97.0 (C_5Me$_5$), 8.7 (C$_5$*Me*$_5$); IR (dichloromethane, cm^{-1}); $\bar{\upsilon}$ = 2506 (B-H_t), 2467 (B-H_t), 2422 (B-H_t).

3: MS (ESI$^+$): *m/z* calculated for $[C_{10}H_{24}B_4Os + CH_3CN + H]^+$:419.2210, found: 419.2204; ^{1}H NMR (500 MHz, C_6D_6, 22 °C): δ = 2.98 (br, B-H$_t$), 2.00 (br, B-H$_t$); 1.56 (s, 15H, 1×Cp*), −3.79 (br, 1H, B-*H*-B), −4.13 (br, 2H, B-*H*-B), −15.60 (br, 2H, Os-*H*-B); ^{11}B{^{1}H} NMR (160 MHz, C_6D_6, 22 °C): *δ* (ppm) = −2.5 (br, 1B), −12.1 (br, 1B); ^{1}H{^{11}B} NMR (500 MHz, C_6D_6, 22 °C): *δ* (ppm)= 2.98 (s, 2H, B-H$_t$), 2.01 (s, 2H, B-H$_t$); 1.56 (s, 15H, 1×Cp*), −3.79 (s, 1H, B-*H*-B), −4.16 (s, 2H, B-*H*-B), −15.61 (s, 2H, Os-*H*-B); ^{13}C{^{1}H} NMR (125 MHz, C_6D_6, 22 °C): *δ* (ppm) = 95.7 (C_5Me$_5$), 9.2 (C$_5$*Me*$_5$); IR (dichloromethane, cm^{-1}): $\bar{\upsilon}$ = 2513 (B-H_t), 2453 (B-H_t), 2411 (B-H_t).

I.1 Spectroscopic details

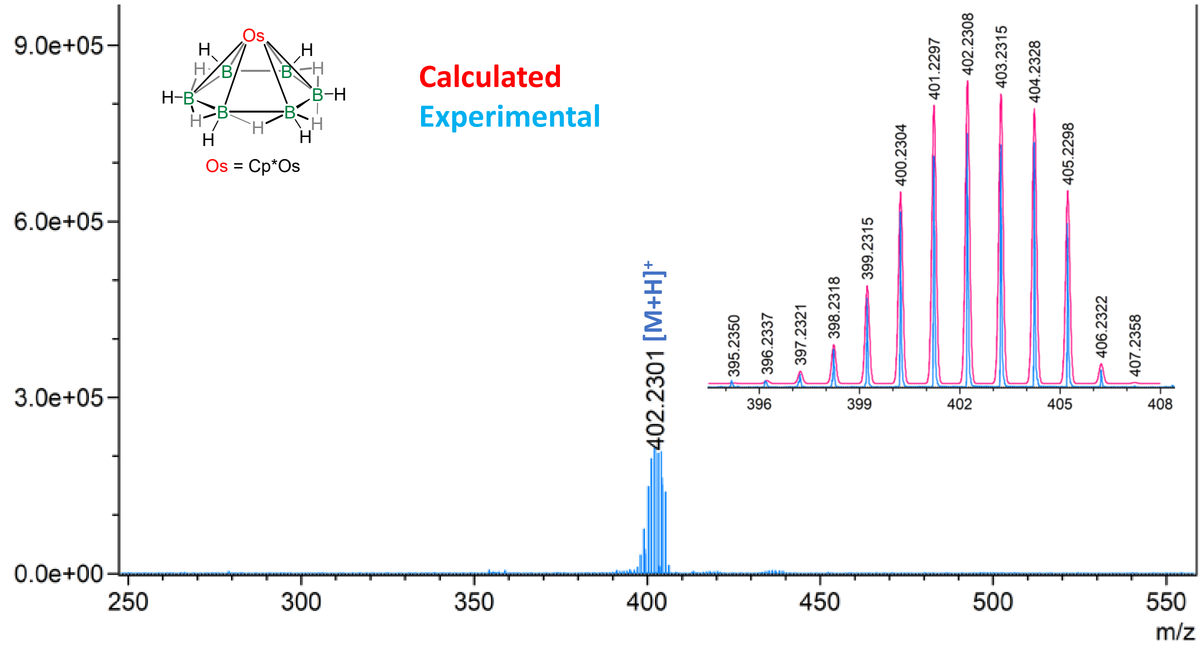

Figure S1. ESI-MS spectrum of **1** in CH_2Cl_2.

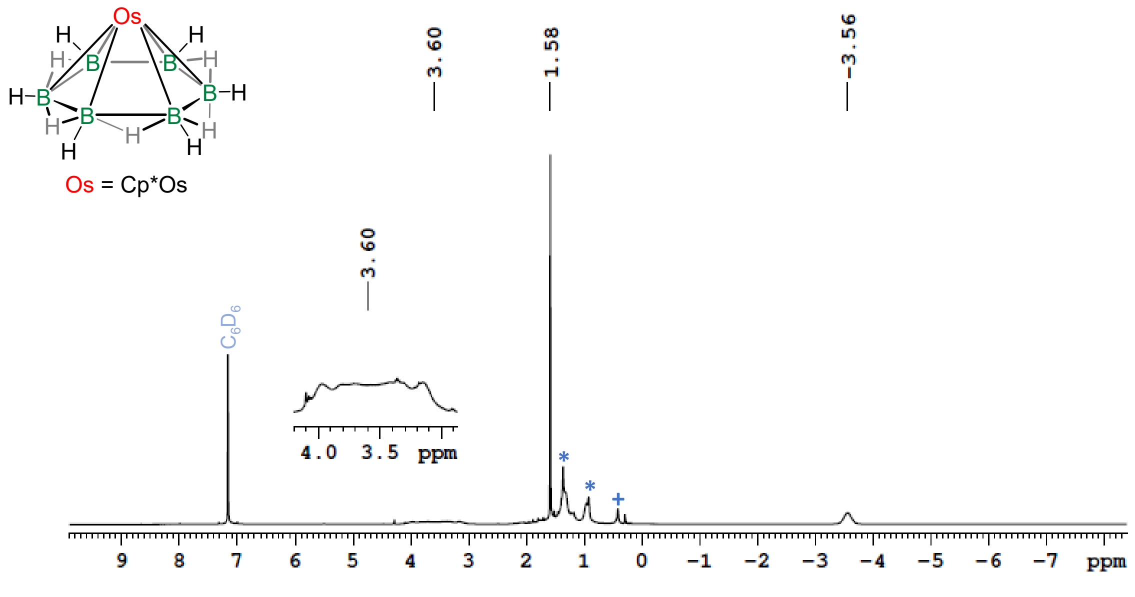

Figure S2. ^{1}H NMR spectrum of **1** in C_6D_6. (+H_2O,*Hexane)

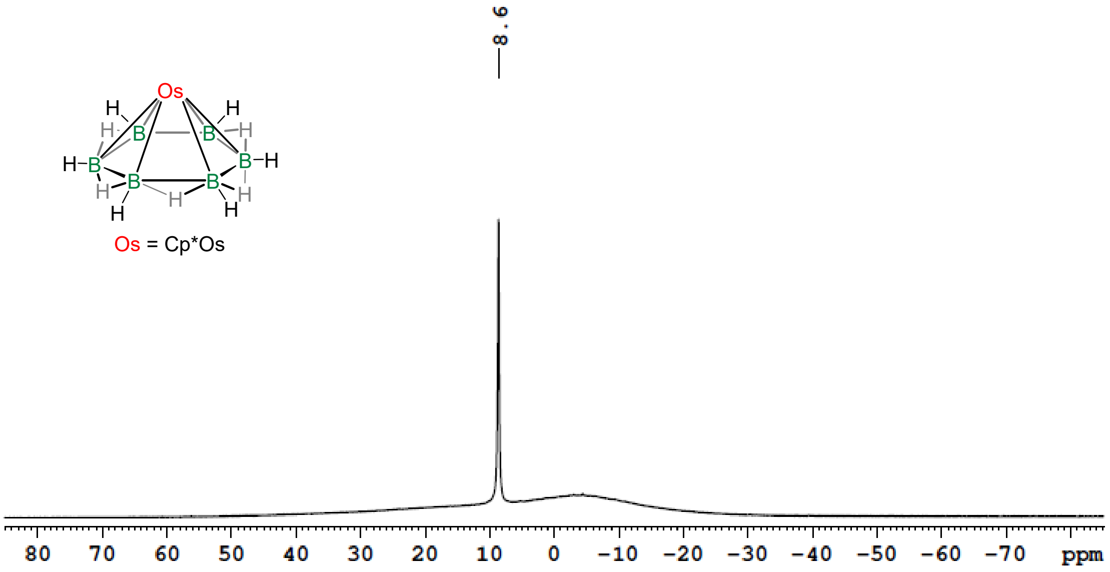

Figure S3. $^{11}B\{^{1}H\}$ NMR spectrum of **1** in C_6D_6.

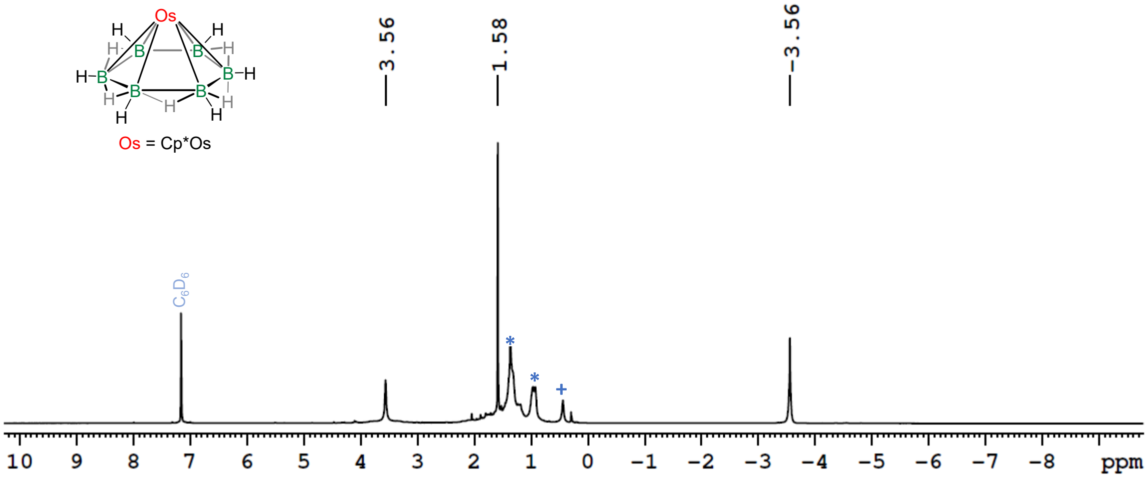

Figure S4. $^{1}H\{^{11}B\}$ NMR spectrum of **1** in C_6D_6. (+H_2O, *Hexane)

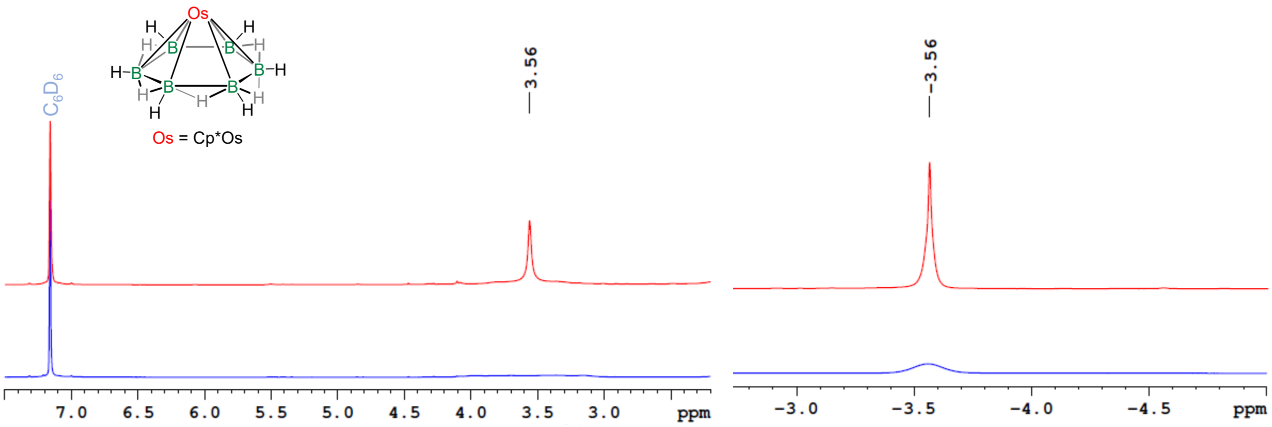

Figure S5. Stacked ^{1}H (blue) and $^{1}H\{^{11}B\}$ (red) NMR spectra of **1** in C_6D_6.

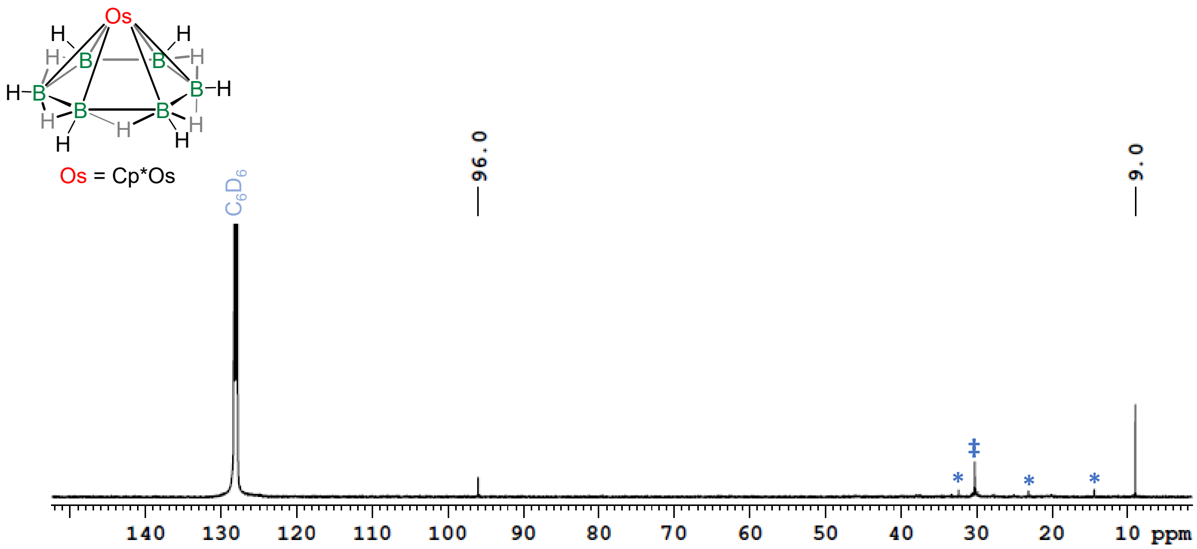

Figure S6. $^{13}C\{^{1}H\}$ NMR spectrum of **1** in C_6D_6. (‡H-Grease,*Hexane)

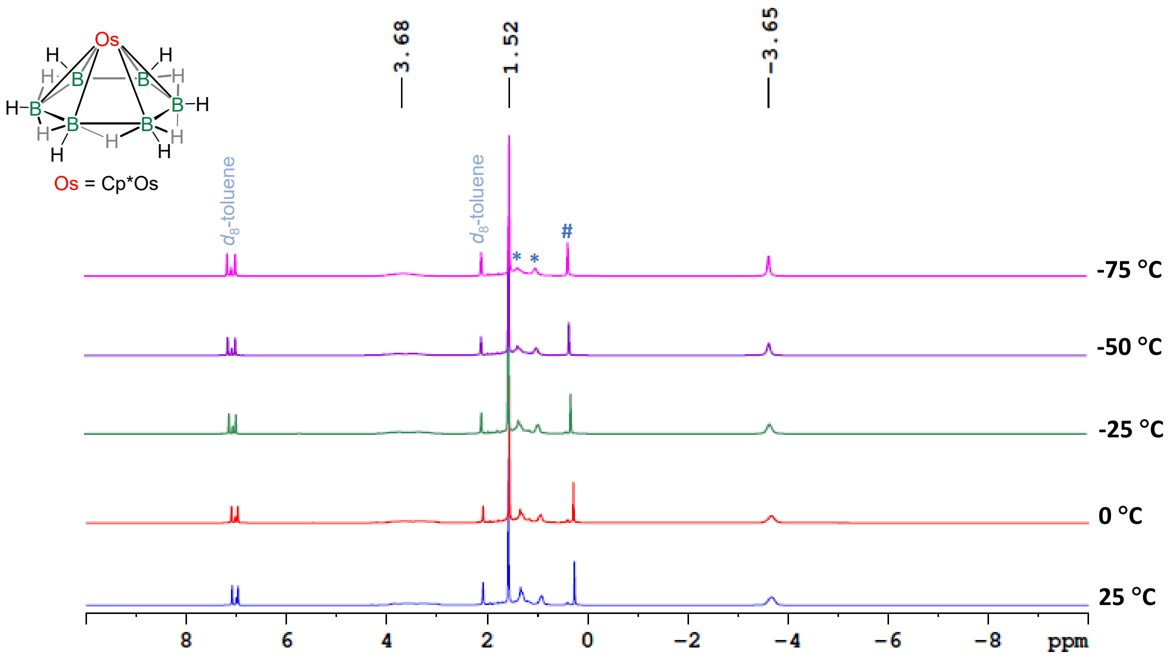

Figure S7. Variable temperature ^{1}H NMR spectra of **1** in d_8-toluene. (#Silicon grease, *Hexane)

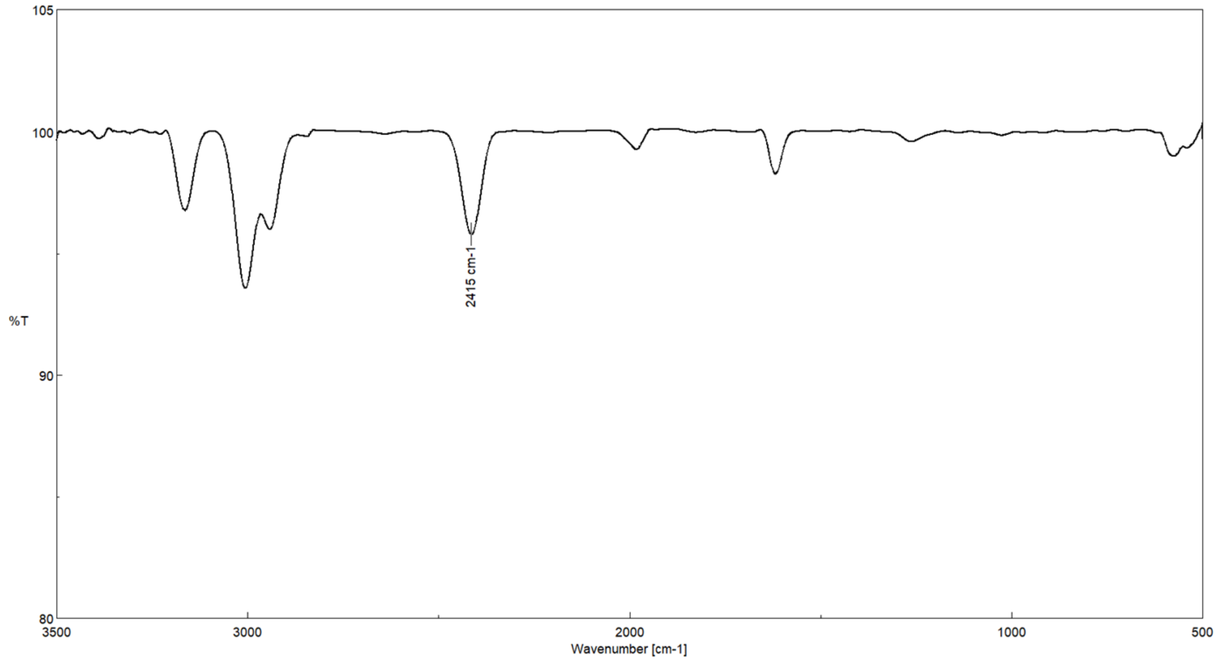

Figure S8. IR spectrum of **1** in CH_2Cl_2.

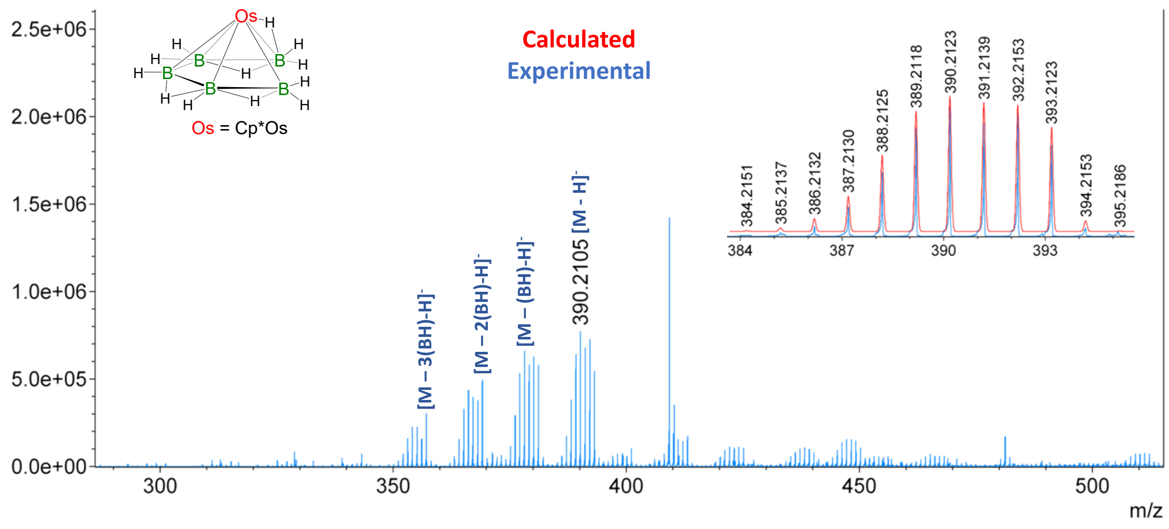

Figure S9. ESI-MS spectrum of **2** in CH_2Cl_2.

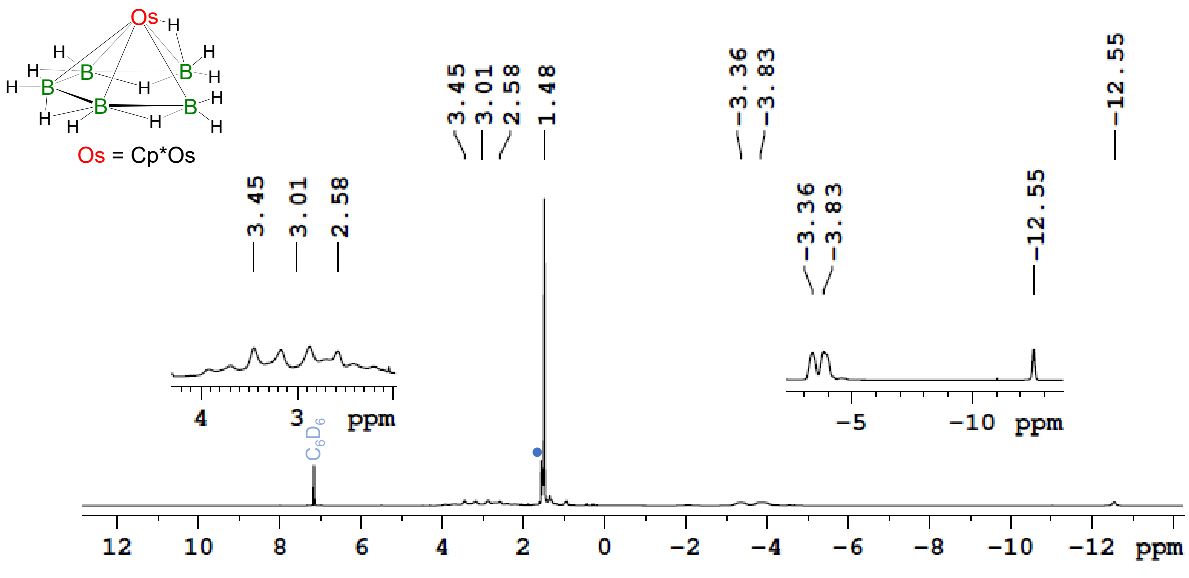

Figure S10. ^{1}H NMR spectrum of **2** in C_6D_6. (•peak due to the presence of complex **3**)

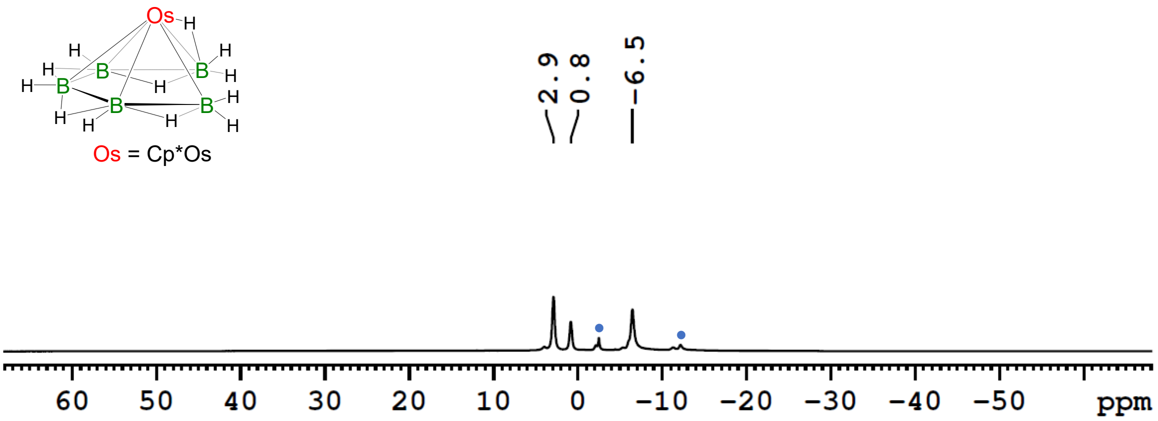

Figure S11. $^{11}B\{^{1}H\}$ NMR spectrum of **2** in C_6D_6. (•peak due to the presence of complex **3**)

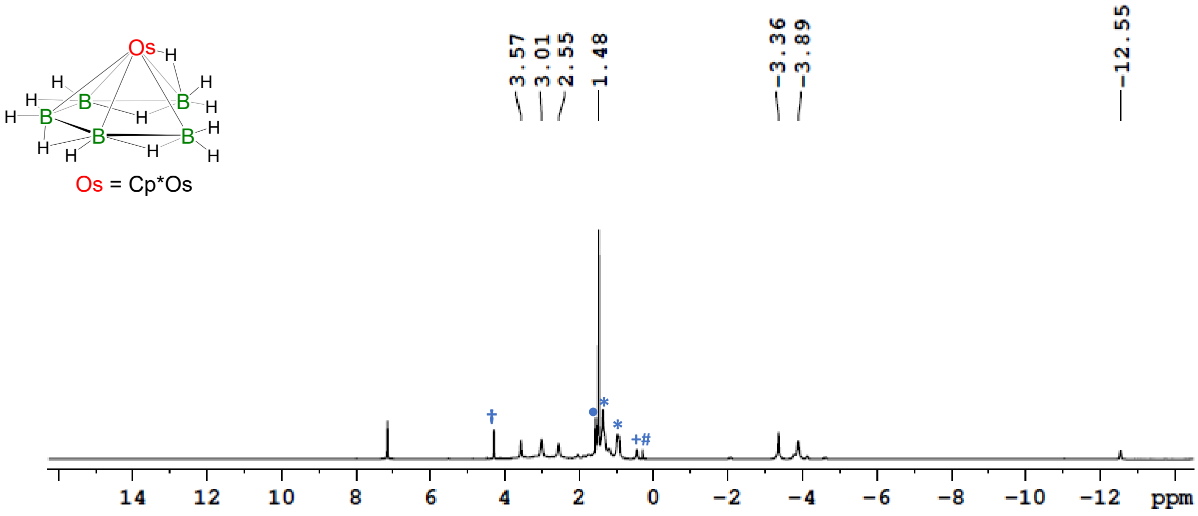

Figure S12. $^{1}H\ \{^{11}B\}$ NMR spectrum of **2** in C_6D_6. (+H_2O, #Silicon grease, *Hexane †CH_2Cl_2,•peak due to the presence of complex **3**)

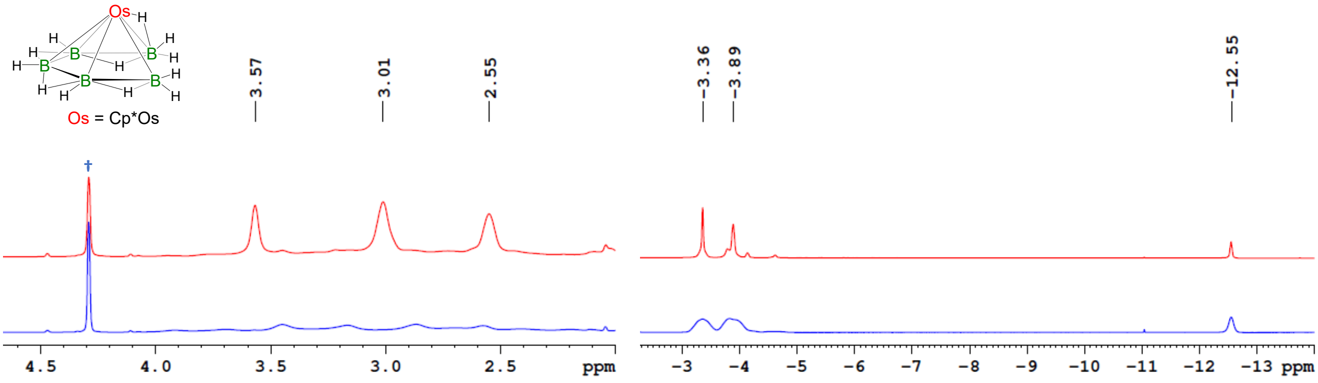

Figure S13. Stacked ^{1}H (blue) and $^{1}H\{^{11}B\}$ (red) NMR spectra of **2** in C_6D_6**.** (†CH_2Cl_2)

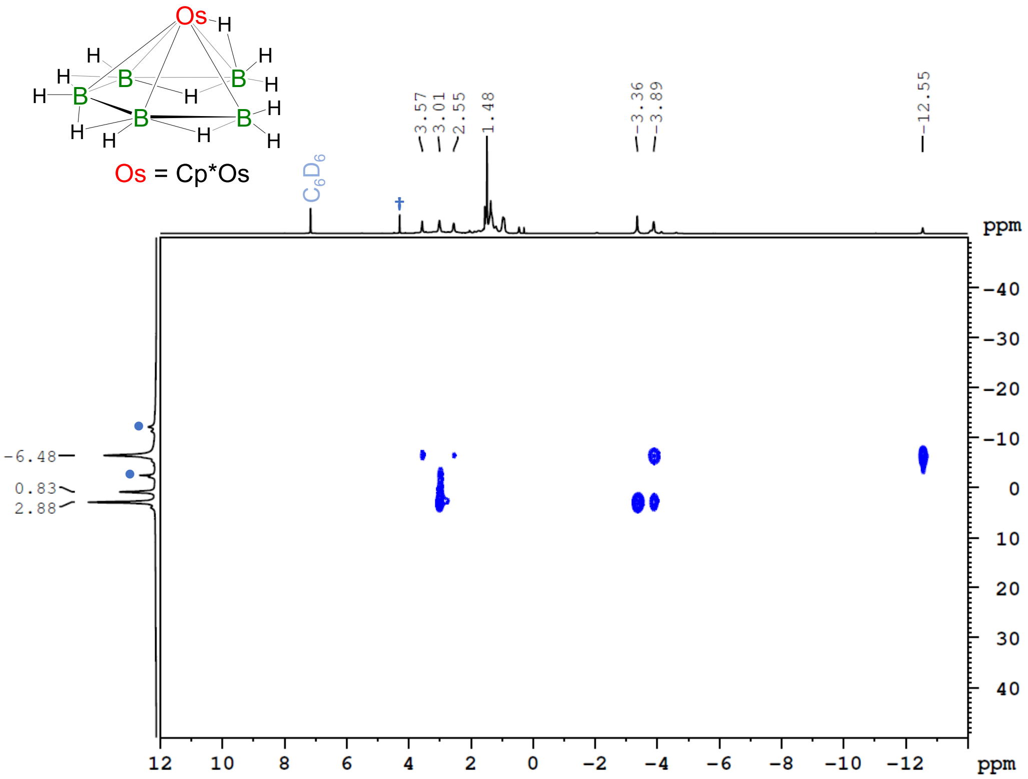

Figure S14. ^{1}H-^{11}B HSQC NMR spectrum of **2** in C_6D_6. (•peak due to the presence of complex **3**, †CH_2Cl_2)

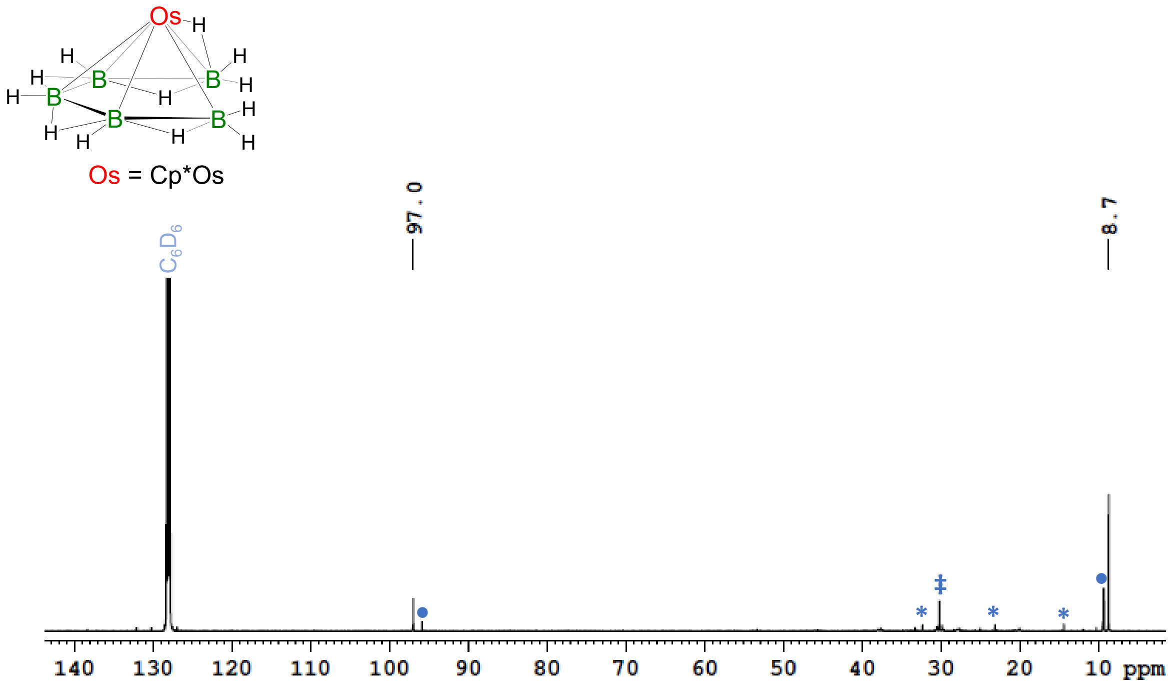

Figure S15. $^{13}C\{^1H\}$ NMR spectrum of **2** in C_6D_6. (‡H-Grease, *Hexane, •peak due to the presence of complex **3**)

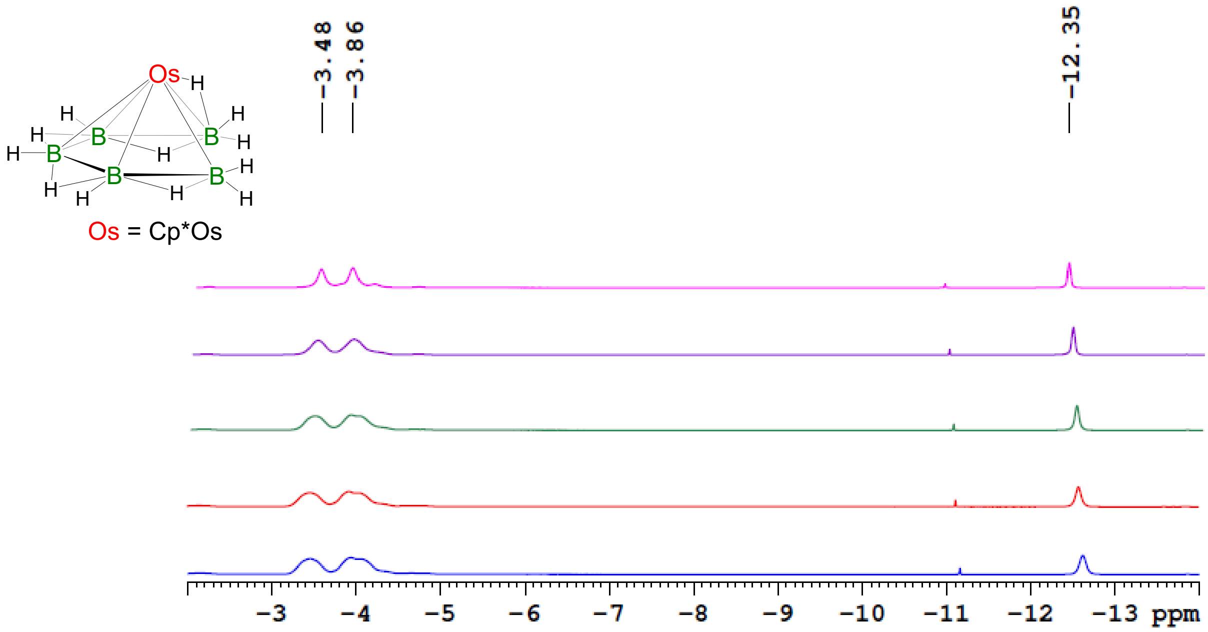

Figure S16. Variable temperature ^1H NMR spectra (hydride region) of **2** in d_8-toluene.

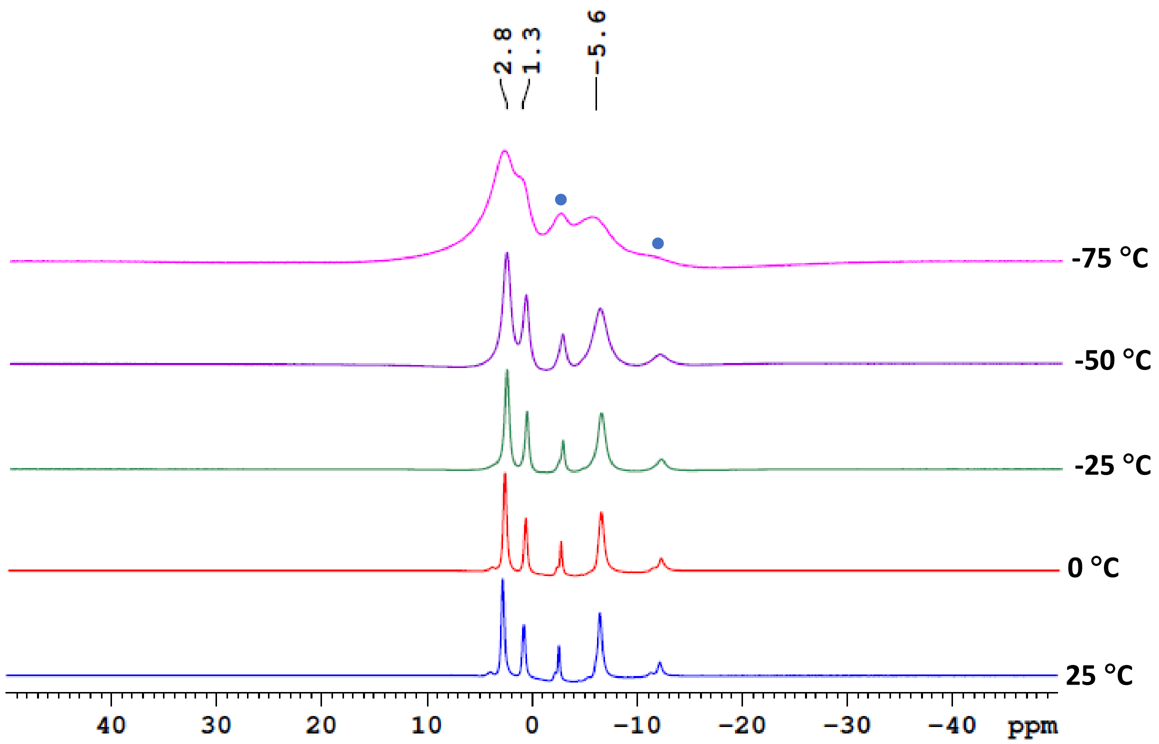

Figure S17. Variable temperature $^{11}B\{^{1}H\}$ NMR spectra of **2** in d_8-toluene. (●peak due to the presence of complex **3**)

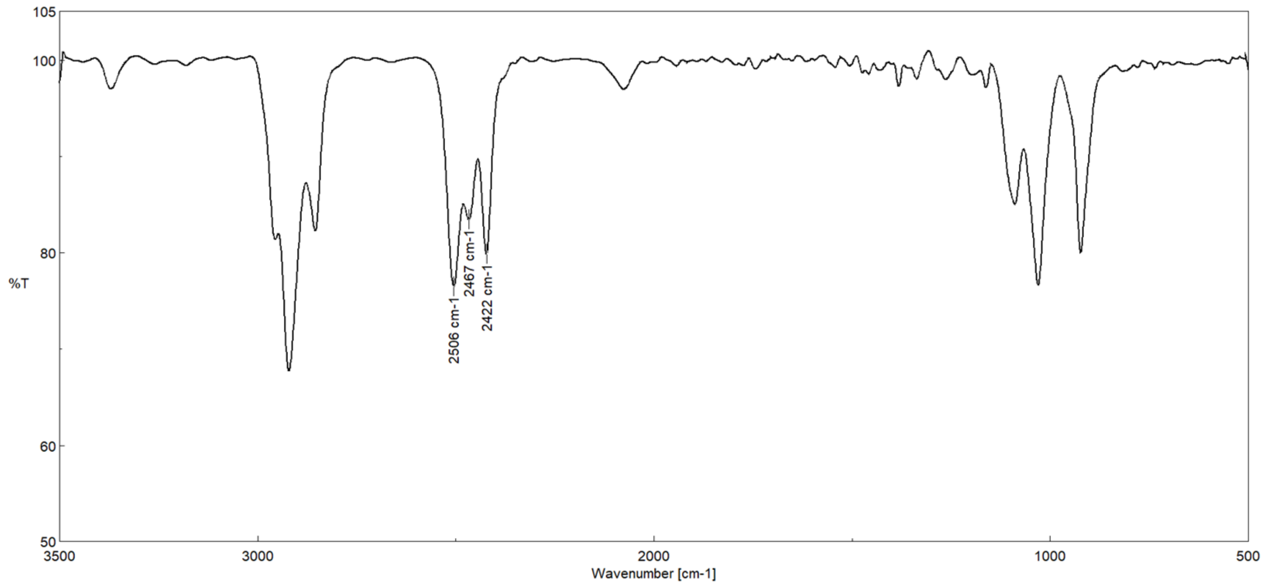

Figure S18. IR spectrum of **2** in CH_2Cl_2.

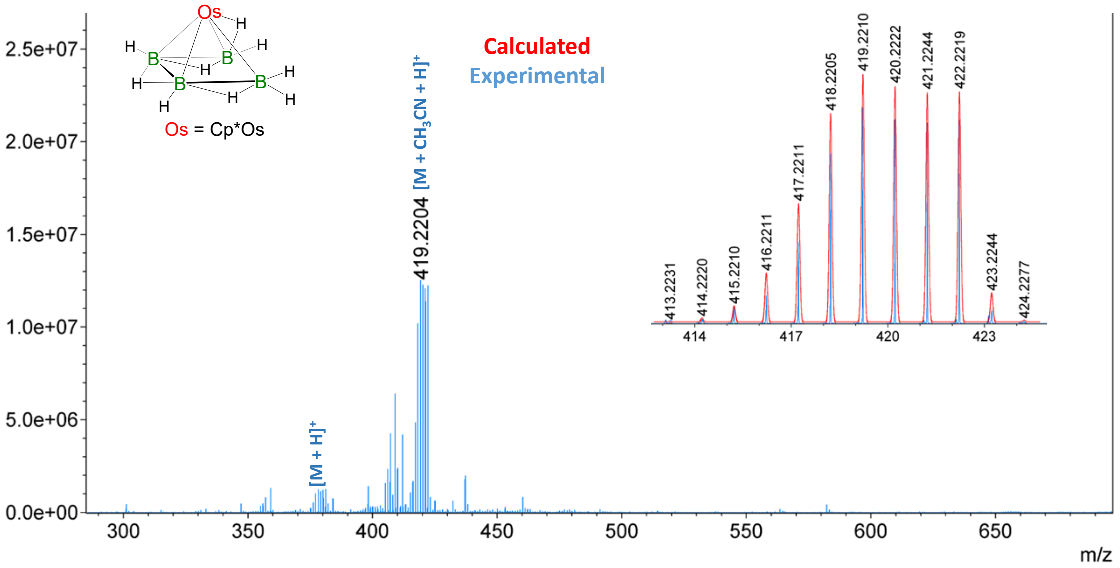

Figure S19. ESI-MS spectrum of **3** in CH_2Cl_2.

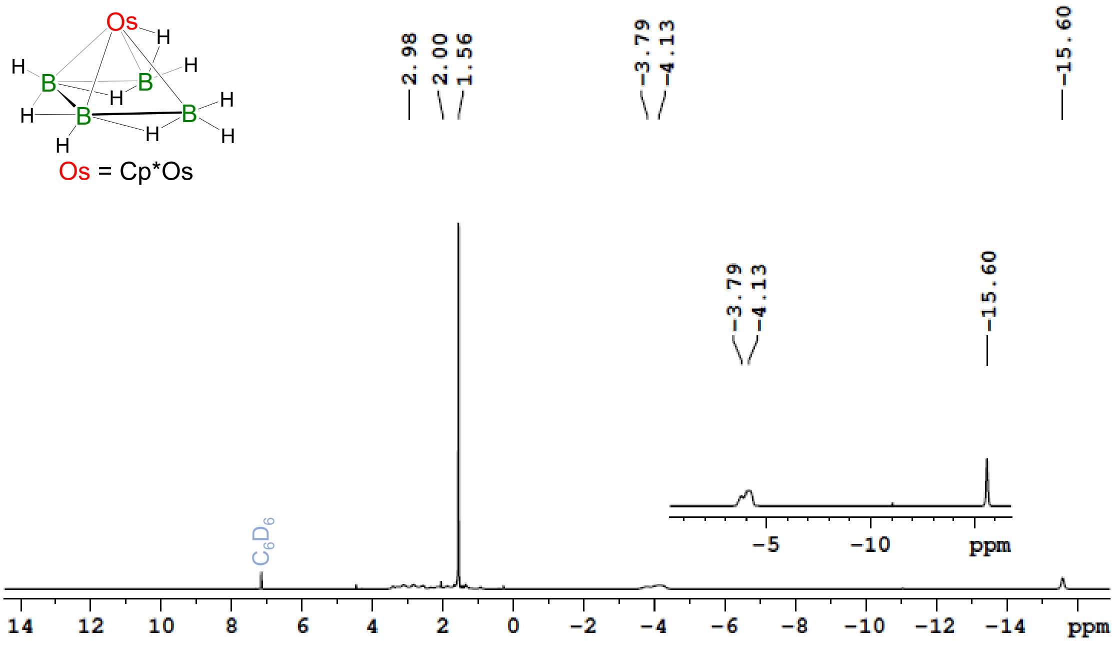

Figure S20. ^{1}H NMR spectrum of **3** in C_6D_6.

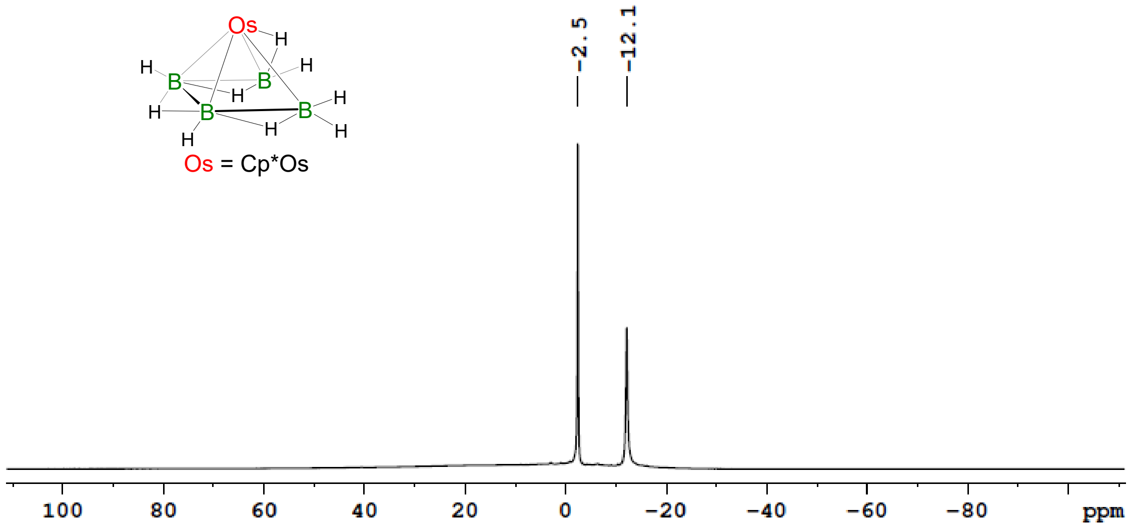

Figure S21. $^{11}B\{^{1}H\}$ NMR spectrum of **3** in C_6D_6.

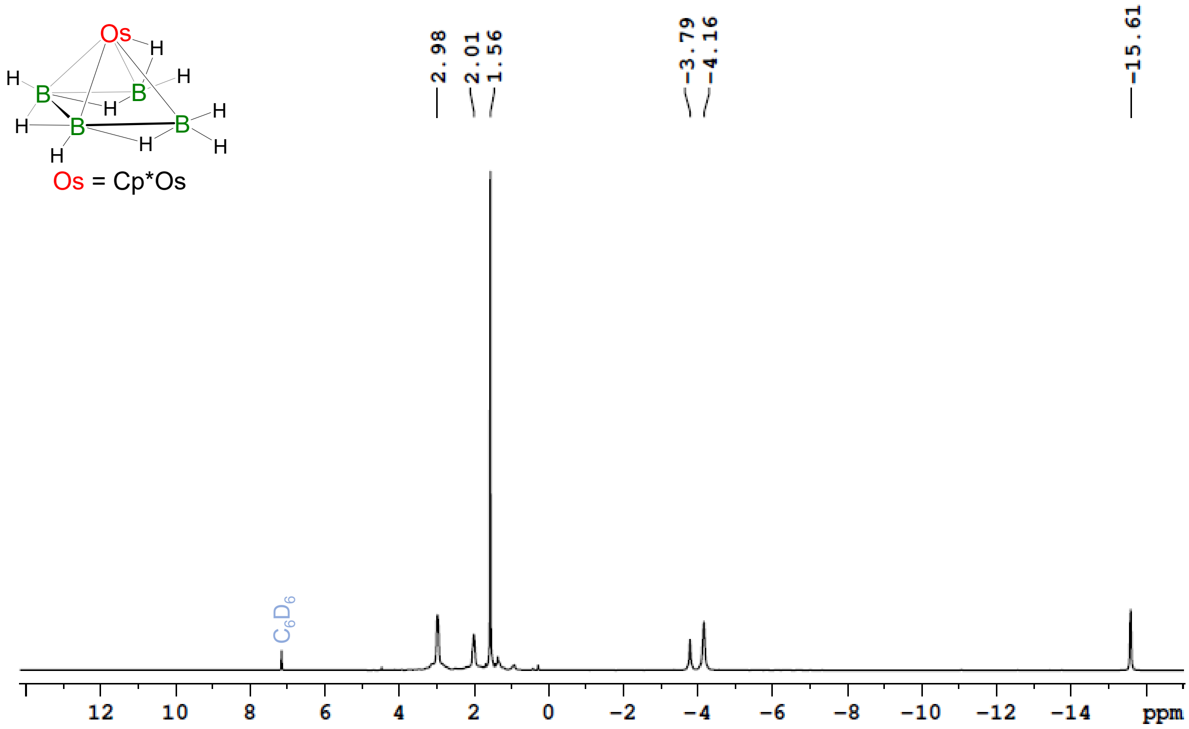

Figure S22. $^{1}H\{^{11}B\}$ NMR spectrum of **3** in C_6D_6.

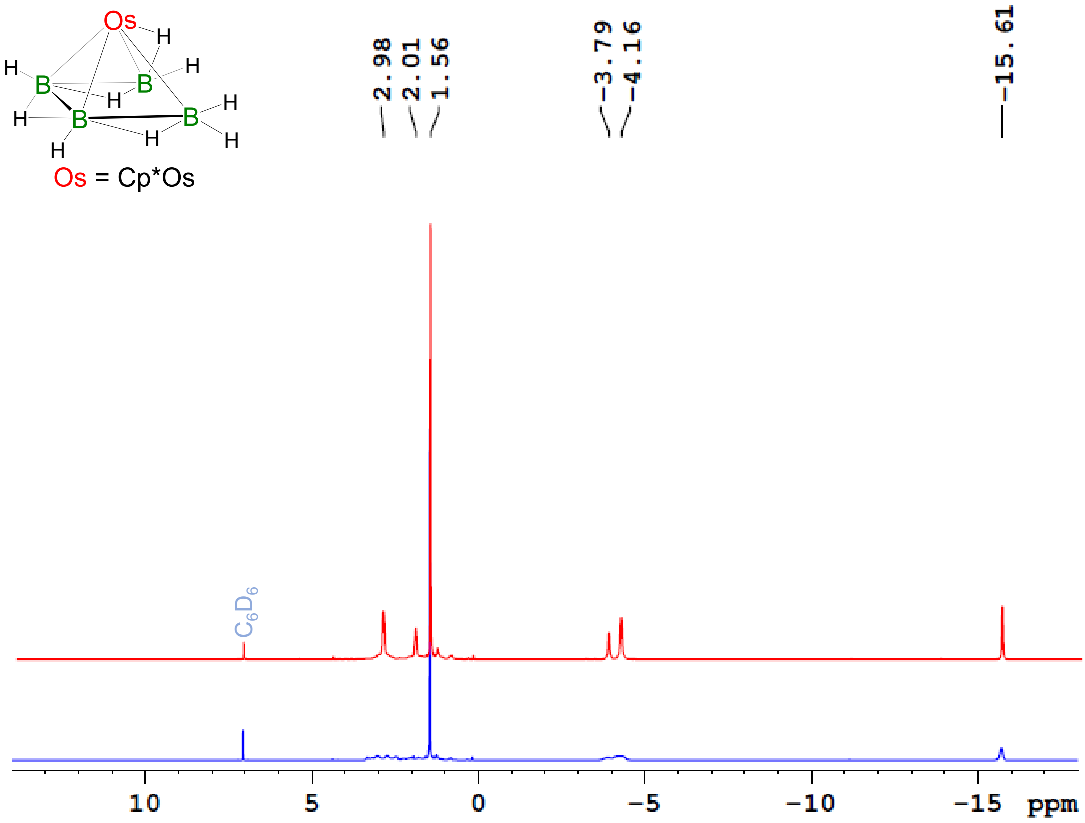

Figure S23. Stacked ^{1}H (blue) and $^{1}H\{^{11}B\}$ (red) NMR spectra of **3** in C_6D_6**.**

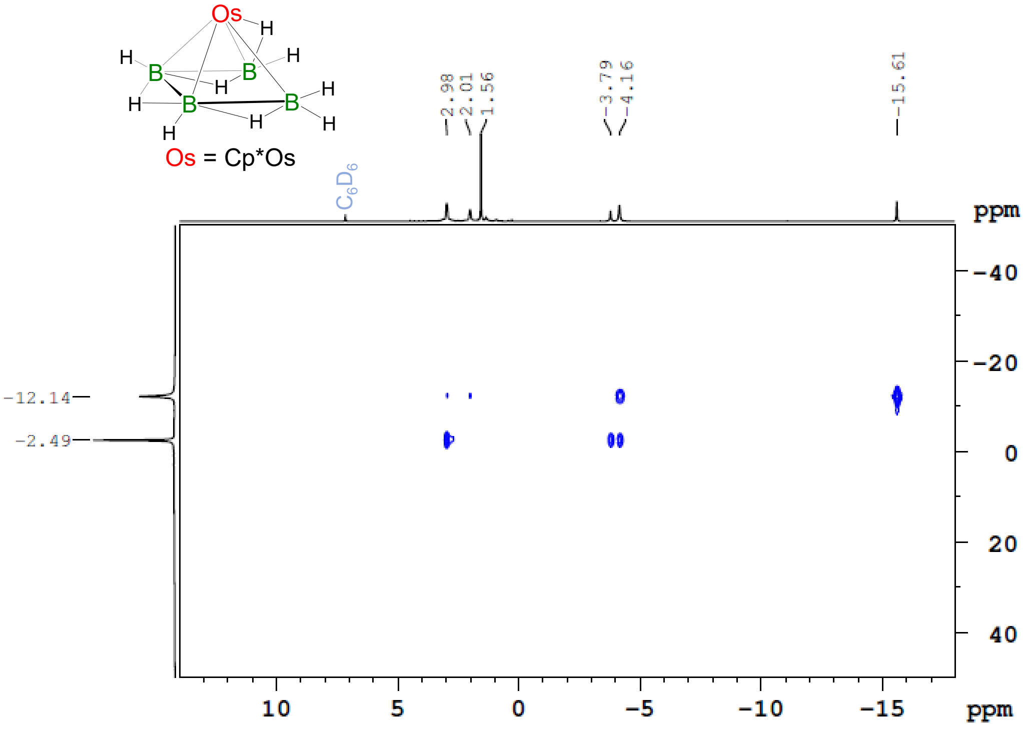

Figure S24. ^{1}H-^{11}B HSQC NMR spectrum of **3** in C_6D_6.

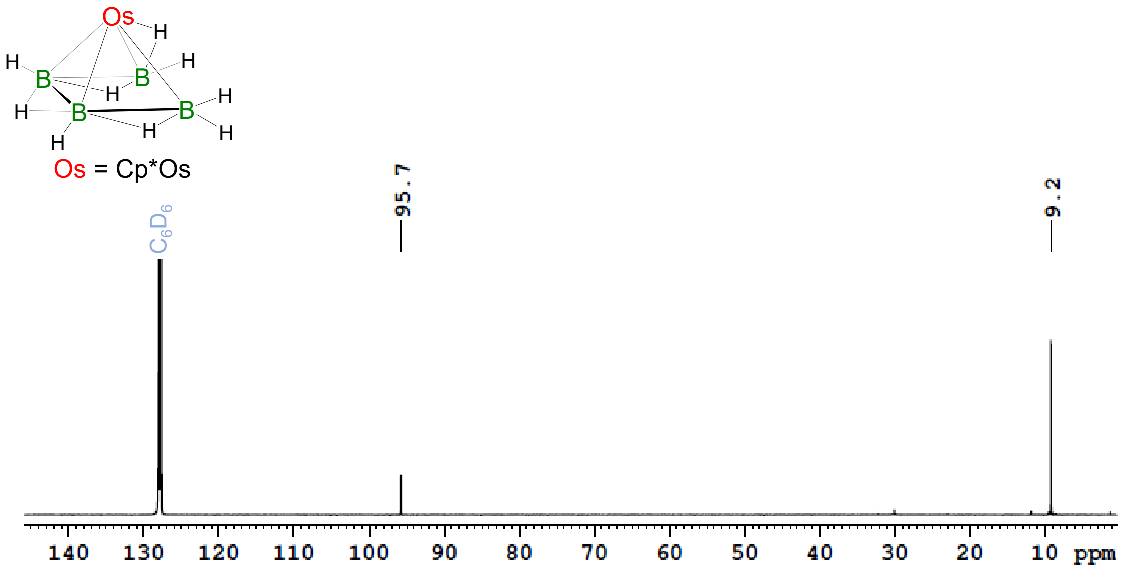

Figure S25. $^{13}C\{^{1}H\}$ NMR spectrum of **3** in C_6D_6.

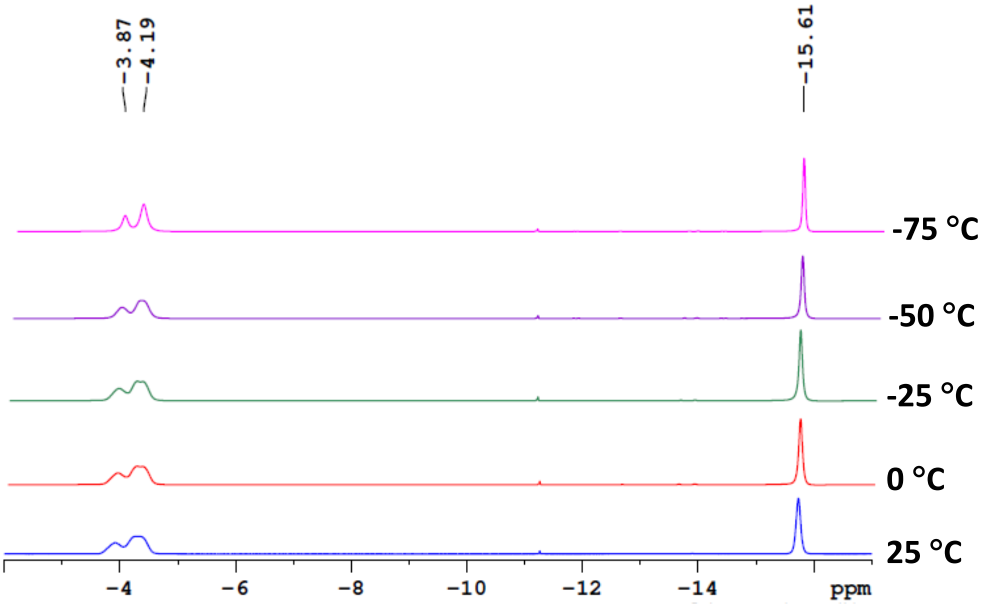

Figure S26. Variable temperature ^{1}H NMR spectra (Hydride region) of **3** in d_8-toluene.

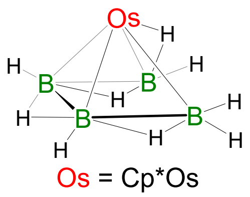

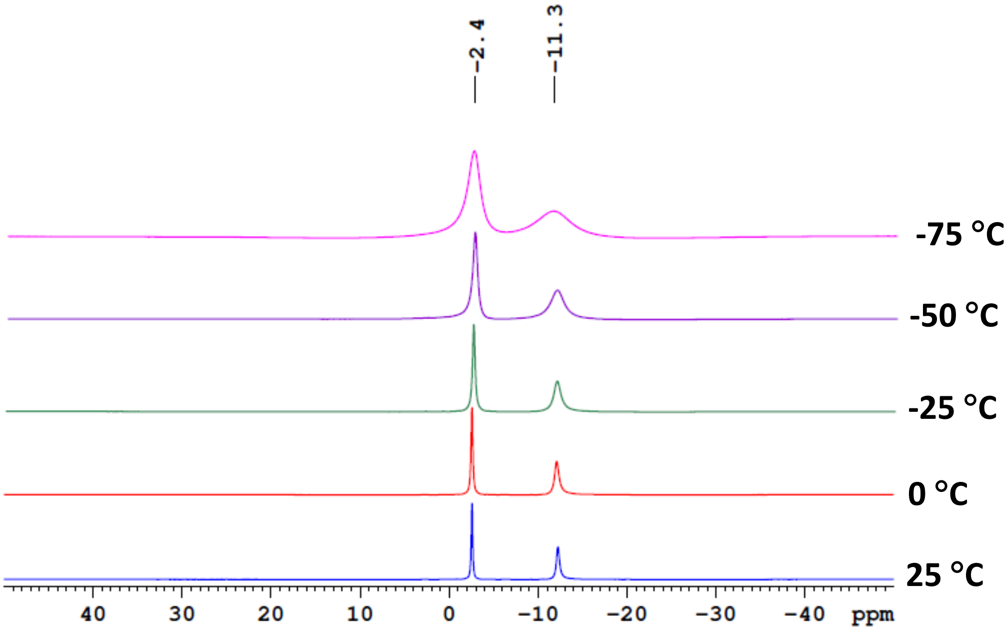

Figure S27. Variable temperature $^{11}B\{^{1}H\}$ NMR spectra of **3** in d_8-toluene.

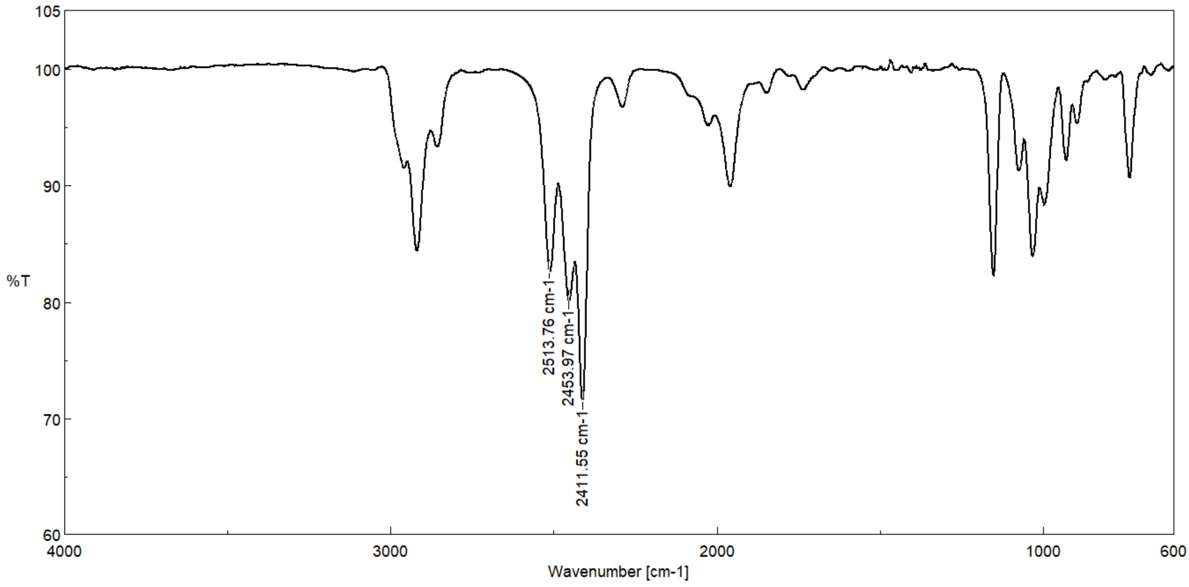

Figure S28. IR spectrum of **3** in CH_2Cl_2.

I.2 X-ray Analysis Details

Suitable X-ray quality crystals of **1**, **2** and **3** were grown by slow diffusion of a hexane-CH_2Cl_2 solution at -4 ˚C. Crystal data of **1** was obtained and integrated using a Bruker D8 VENTURE diffractometer with PHOTON II detector with graphite monochromated Mo-Kα (λ = 0.71073 Å) radiation at 100(2) K. **2** was obtained and integrated using a Bruker Kappa Apex2 CCD diffractometer with graphite monochromated Mo-Kα (λ = 0.71073 Å) radiation at 110(2) K whereas **3** was obtained and integrated using a Bruker Kappa Apex3 CMOS diffractometer with graphite monochromated Mo-Kα (λ = 0.71073 Å) radiation at 150(2) K. All the structures were solved using SHELXT-2018 and SHELXS-97[4,5] and refined using SHELXL–2018, SHELXL-2014 and SHELXL-2019.[6] Using Olex2 all the molecular structures were drawn.[7] Crystallographic data have been deposited with the Cambridge Crystallographic Data Centre as supplementary publication no CCDC- 2438685 (**1**), 2438887 (**2**), 2438789 (**3**). These data can be obtained free of charge from The Cambridge Crystallographic Data Centre via www.ccdc.cam.ac.uk/data_request/cif.

Crystal data for **1**: $C_{10}H_{26}B_6Os$, M_r = 401.37, Monoclinic, space group *P 21*, a = 7.1489(2) Å, b = 13.4533(4) Å, c = 8.2583(2) Å, α = 90°, β = 108.2260(10)°, γ = 90°, V = 754.41(4) Å^3, Z = 2, ρ_{calcd} = 1.771 g/cm^3, μ = 8.423 mm^{-1}, $F(000)$ = 386.0, R_1 = 0.0215, wR_2 = 0.0503, 3596 independent reflections [2θ ≤ 55.814°] and 304 parameters.

Crystal data for **2**: $C_{10}H_{27}B_5Os$, M_r = 391.56, Orthorhombic, space group *P n a 21*, a = 13.9545(5) Å, b = 8.2607(3) Å, c = 13.1269(5) Å, α = 90°, β = 90°, γ = 90°, V = 1513.19(10) Å^3, Z = 4, ρ_{calcd} = 1.719 g/cm^3, μ = 8.397 mm^{-1}, $F(000)$ = 752.0, R_1 = 0.0453, wR_2 = 0.01260, 3394 independent reflections [2θ ≤ 55.056°] and 297 parameters.

Crystal data for **3**: $C_{10}H_{24}B_4Os$, M_r = 377.73, Orthorhombic, space group *P 21 21 21*, a = 8.1325(3) Å, b = 12.8806(5) Å, c = 14.1056(4) Å, α = 90°, β = 90°, γ = 90°, V = 1477.58(9) Å^3, Z = 4, ρ_{calcd} = 1.698 g/cm^3, μ = 8.597 mm^{-1}, $F(000)$ = 720.0, R_1 = 0.0143, wR_2 = 0.0354, 2895 independent reflections [2θ ≤ 51.99°] and 186 parameters.

II Computational Details

All computational calculations were performed using the Gaussian 16 software (Revision C.01).[8] The analysis is based on results obtained with the B3LYP functional (Becke exchange combined with Hartree-Fock and the Lee-Yang-Parr correlation functional),[9-11] with empirical dispersion corrections (GD3).[12] The Def2-SVP basis set[13,14] was applied to all atoms. Solvent effects were modelled using the polarisable continuum model (PCM) with the integral

equation formalism (IEFPCM)[15] for toluene. All structures were verified as either minima or transition states by checking for zero or one imaginary frequency, respectively. Molecular visualizations were carried out using Chemcraft.[16] The localized orbital picture is generated by IBO analysis in IBOView software.[17,18]

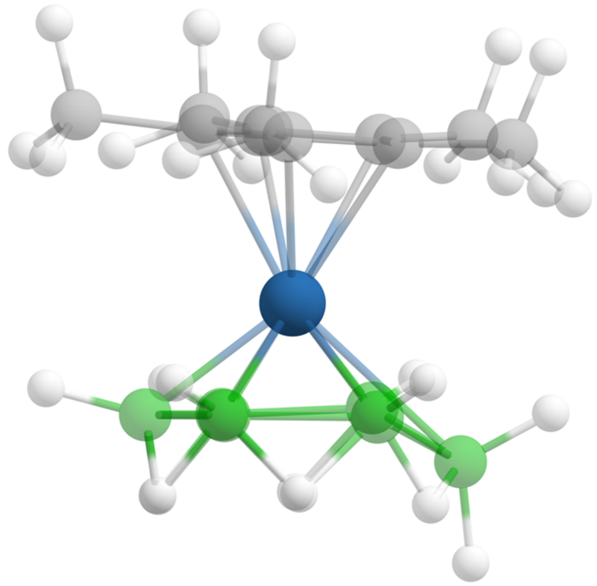

Figure S29. Optimized geometry of [Cp*Os(η^6-B_6H_{12})] in doublet state at B3LYP-D3/Def2-SVP level of theory with implicit solvation.

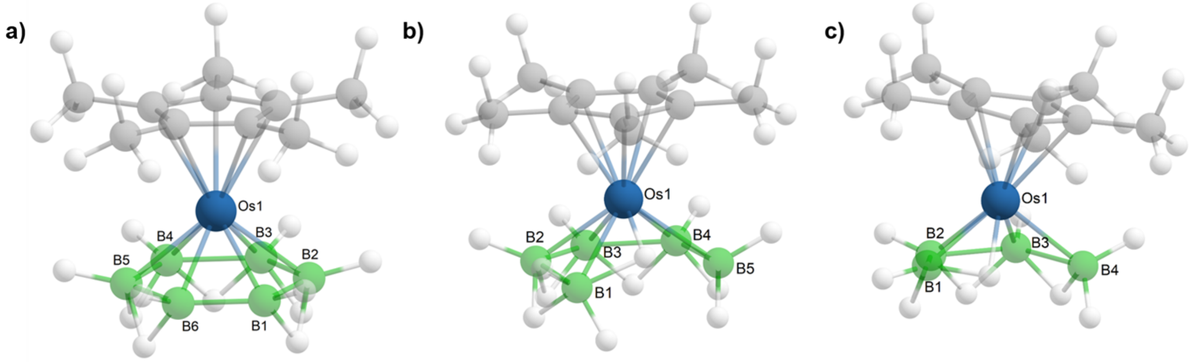

Figure S30. Optimized geometry of a) [Cp*Os(η^6-B_6H_{11})], b) [Cp*Os(η^5-B_5H_{12})], and c) [Cp*Os(η^4-B_4H_9)] at B3LYP-D3/Def2-SVP level of theory with implicit solvation.

Table S1. Bond parameters of complexes **1**-**3** are compared with their optimized values and corresponding WBIs are reported at B3LYP-D3/Def2-SVP level of theory with implicit solvation. All distances are in Å. Labelling is followed from Figure S31.

[Cp*Os(B_6H_{11})] (**1**)			
	Expt.	Cal.	WBI
**Os1-B1**	2.24(3)	2.278	0.35
**Os1-B2**	2.17(19)	2.192	0.42
**Os1-B3**	2.21(2)	2.165	0.44
**Os1-B4**	2.18(3)	2.165	0.44
**Os1-B5**	2.16(3)	2.193	0.41
**Os1-B6**	2.17(3)	2.278	0.35
**B1-B2**	1.756(17)	1.761	0.64
**B2-B3**	1.75(2)	1.808	0.56
**B3-B4**	1.75(2)	1.797	0.57
**B4-B5**	1.764(14)	1.808	0.56
**B5-B6**	1.74(2)	1.761	0.64
**B6-B1**	1.75(2)	1.635	1.09

[Cp*Os(B_5H_{12})] (**2**)			
	Expt.	Cal.	WBI
**Os1-B1**	2.24(3)	2.324	0.32
**Os1-B2**	2.02(3)	2.170	0.44
**Os1-B3**	2.15(3)	2.177	0.42
**Os1-B4**	2.23(3)	2.176	0.42
**Os1-B5**	2.16(3)	2.286	0.42
**B1-B2**	1.77(4)	1.839	0.54
**B2-B3**	1.99(4)	1.801	0.58
**B3-B4**	1.77(4)	1.805	0.57
**B4-B5**	1.74(3)	1.794	0.61

[Cp*Os(B_4H_9)] (**3**)			
	Expt.	Cal.	WBI
**Os1-B1**	2.305(7)	2.015	0.73
**Os1-B2**	2.146(7)	2.196	0.42
**Os1-B3**	2.150(7)	2.157	0.44
**Os1-B4**	2.300(7)	2.238	0.50
**B1-B2**	1.846(11)	1.792	0.61
**B2-B3**	1.826(12)	1.826	0.55
**B3-B4**	1.851(13)	1.811	0.60

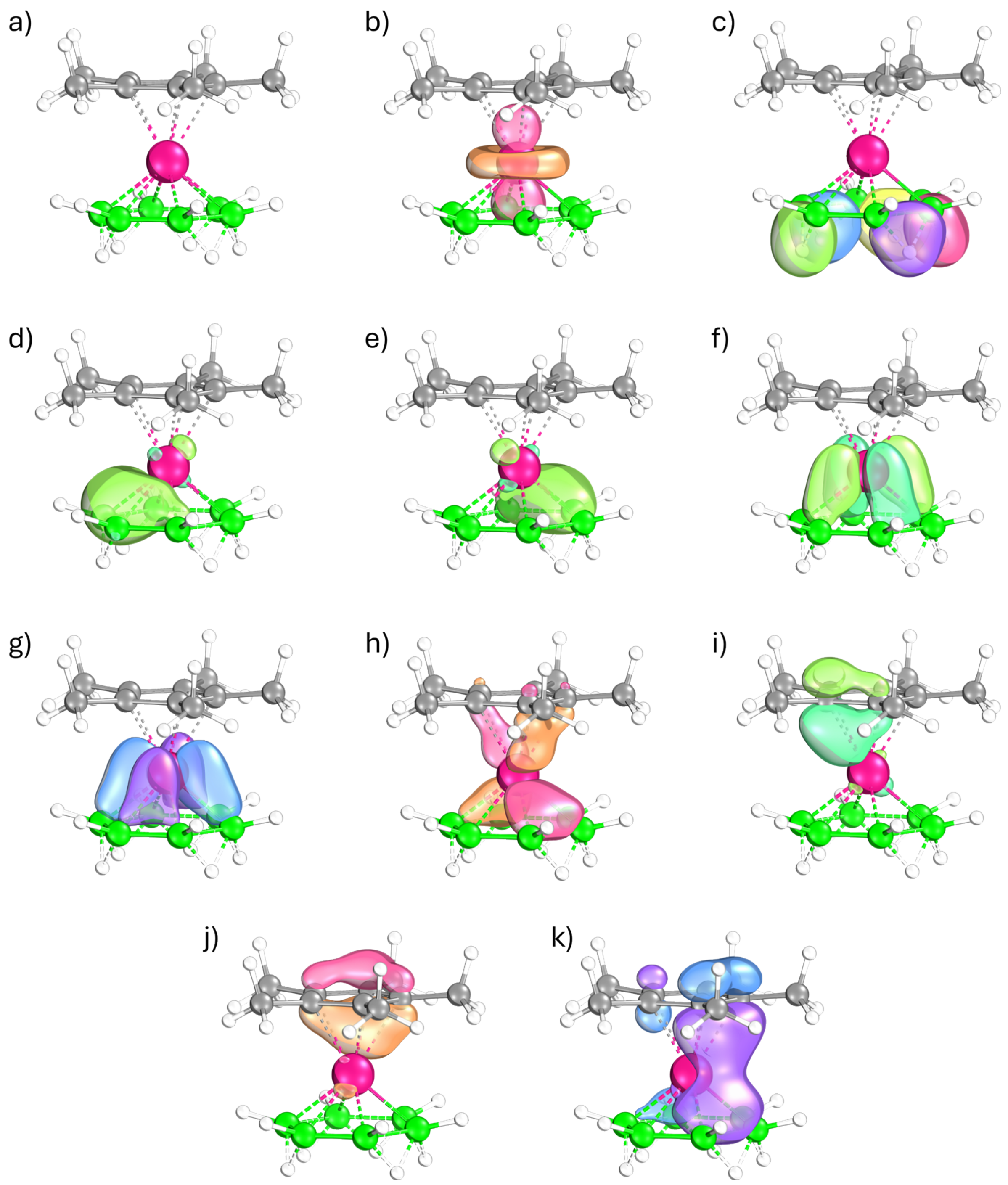

Figure S31. Selected localized orbitals of **1** (a) computed using IBOview, illustrating (b) a 1c-2e lone pair on osmium, (c) five 3c-2e bond orbitals, and (d-k) eight multicenter bond orbitals. For clarity, the thirty-two 2c-2e bond orbitals are not shown.

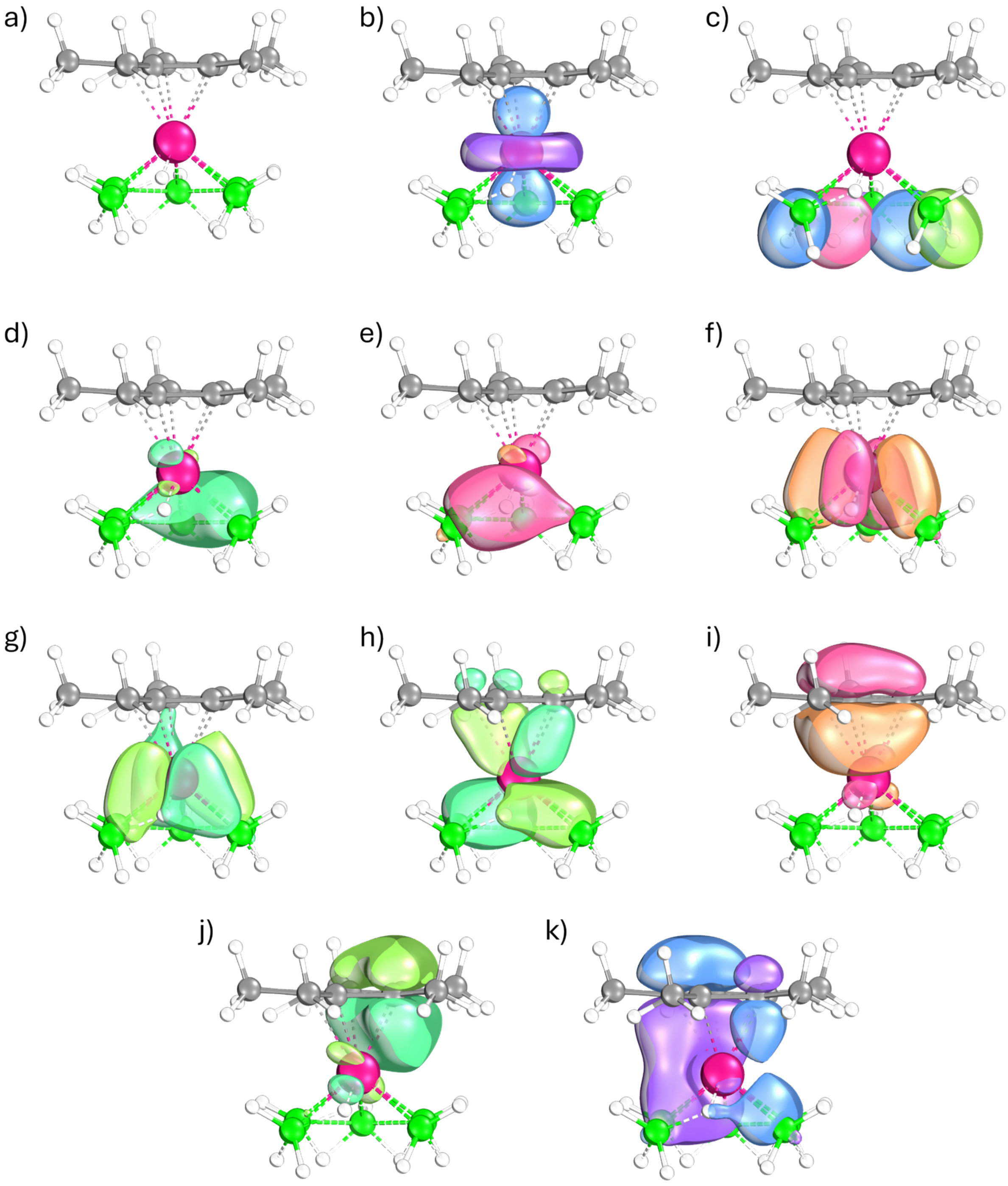

Figure S32. Selected localized orbitals of **2** (a) computed using IBOview, illustrating (b) a 1c-2e lone pair on osmium, (c) four 3c-2e bond orbitals, and (d-k) eight multicenter bond orbitals. For clarity, the thirty-two 2c-2e bond orbitals are not shown.

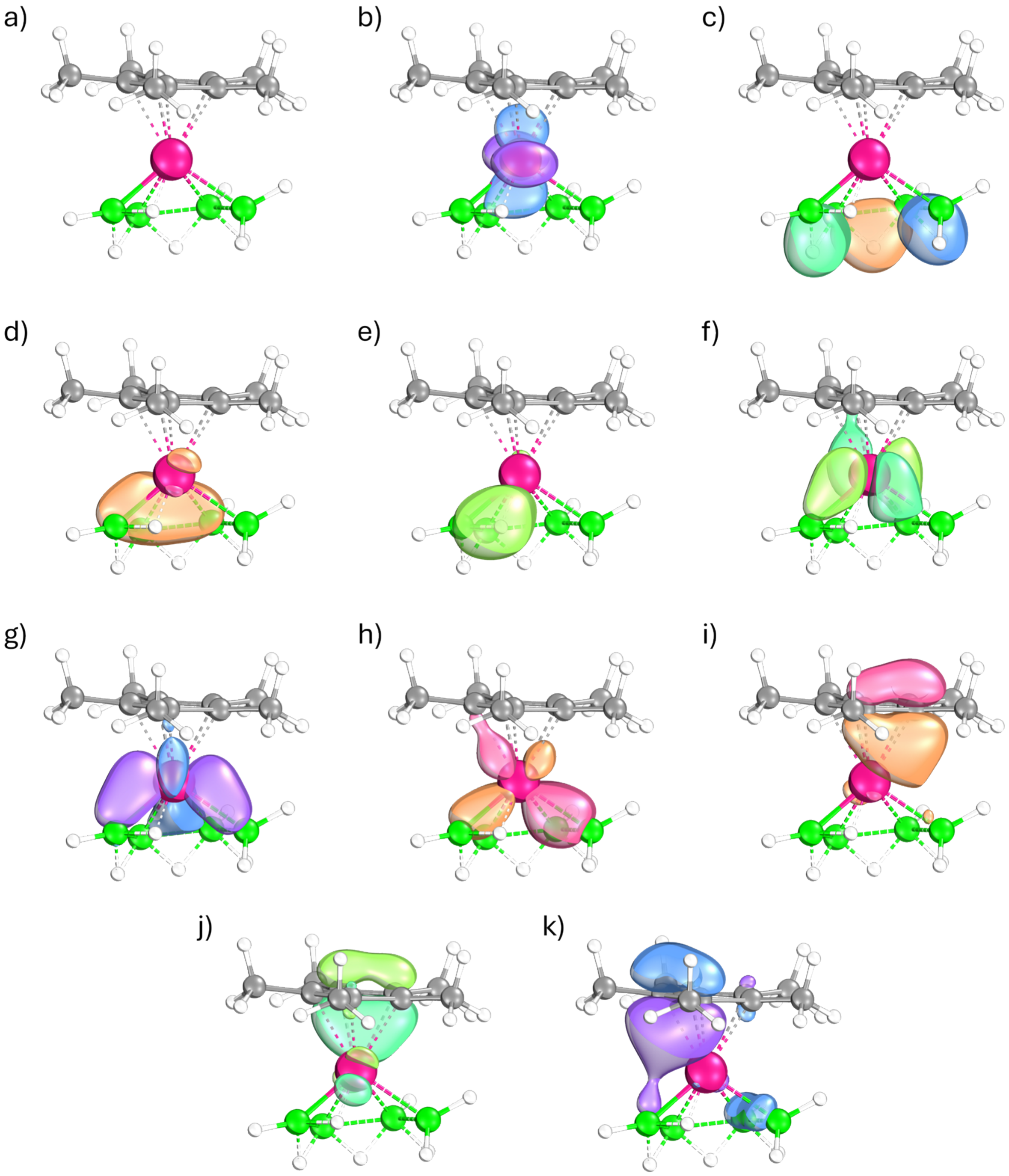

Figure S33. Selected localized orbitals of **3** (a) computed using IBOview, illustrating (b) a 1c-2e lone pair on osmium, (c) three 3c-2e bond orbitals, and (d-k) eight multicenter bond orbitals. For clarity, the thirty 2c-2e bond orbitals are not shown.

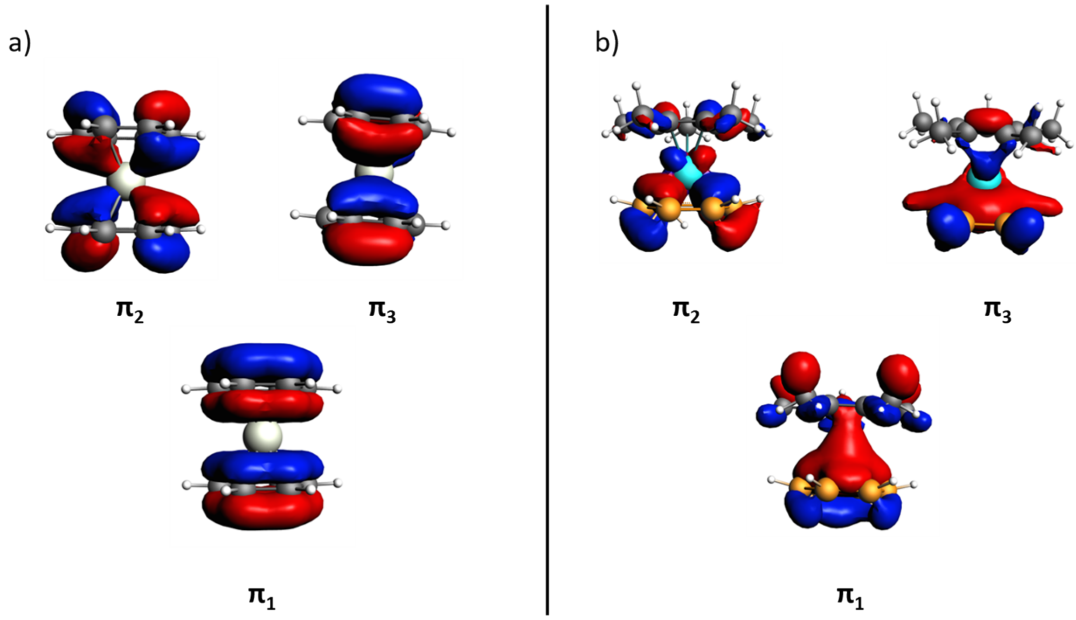

Figure S34. Selected π molecular orbitals of (a) $C_6H_6CrC_6H_6$ and (b) $Cp^*OsB_6H_{11}$ computed at B3LYP-D3/TZP using ADF[18] software.

III References

[1] C. L. Gross, J. L. Brumaghim and G. S. Girolami, *Organometallics*, 2007, **26**, 2258–2265.

[2] J. J. Led and H. Gesmar, *Chem. Rev.*, 1991, **91**, 1413–1426.

[3] R. Weiss and R. N. Grimes, *J. Am. Chem. Soc.*, 1978, **100**, 1401–1405.

[4] G. M. Sheldrick, *Acta Crystallogr., Sect. A: Found. Adv.*, 2015, **71**, 3–8.

[5] G. M. Sheldrick, *SHELXL97*, University of Göttingen, Germany, 1997.

[6] G. M. Sheldrick, *Acta Crystallogr., Sect. C: Struct. Chem.*, 2015, **71**, 3–8.

[7] O. V. Dolomanov, L. J. Bourhis, R. J. Gildea, J. A. K. Howard and H. Puschmann, *J. Appl. Crystallogr.*, 2009, **42**, 339–341.

[8] M. J. Frisch, G. W. Trucks, H. B. Schlegel, G. E. Scuseria, M. A. Robb, J. R. Cheeseman, G. Scalmani, V. Barone, G. A. Petersson, H. Nakatsuji, X. Li, M. Caricato, A. V. Marenich, J. Bloino, B. G. Janesko, R. Gomperts, B. Mennucci, H. P. Hratchian, J. V. Ortiz, A. F. Izmaylov, J. L. Sonnenberg, D. Williams-Young, F. Ding, F. Lipparini, F. Egidi, J. Goings, B. Peng, A. Petrone, T. Henderson, D. Ranasinghe, V. G. Zakrzewski, J. Gao, N. Rega, G. Zheng, W. Liang, M. Hada, M. Ehara, K. Toyota, R. Fukuda, J. Hasegawa, M. Ishida, T. Nakajima, Y. Honda, O. Kitao, H. Nakai, T. Vreven, K. Throssell, J. A. Montgomery Jr., J. E. Peralta, F. Ogliaro, M. J. Bearpark, J. J. Heyd, E. N. Brothers, K. N. Kudin, V. N. Staroverov, T. A. Keith, R. Kobayashi, J. Normand, K. Raghavachari, A. P. Rendell, J. C. Burant, S. S. Iyengar, J. Tomasi, M. Cossi, J. M. Millam, M. Klene, C. Adamo, R. Cammi, J. W. Ochterski, R. L. Martin, K. Morokuma, O. Farkas, J. B. Foresman and D. J. Fox, *Gaussian 16, Revision C.01*, Gaussian, Inc., Wallingford CT, 2016.

[9] A. D. Becke, *J. Chem. Phys.*, 1993, **98**, 5648–5652.

[10] C. Lee, W. Yang and R. G. Parr, *Phys. Rev. B: Condens. Matter Mater. Phys.*, 1988, **37**, 785–789.

[11] P. J. Stephens, F. J. Devlin, C. F. Chabalowski and M. J. Frisch, *J. Phys. Chem.*, 1994, **98**, 11623–11627.

[12] S. Grimme, J. Antony, S. Ehrlich and H. Krieg, *J. Chem. Phys.*, 2010, **132**, 154104.

[13] F. Weigend and R. Ahlrichs, *Phys. Chem. Chem. Phys.*, 2005, **7**, 3297–3305.

[14] F. Weigend, *Phys. Chem. Chem. Phys.*, 2006, **8**, 1057–1065.

[15] J. Tomasi, B. Mennucci and R. Cammi, *Chem. Rev.*, 2005, **105**, 2999–3094.

[16] Chemcraft, Version 1.8 (build 682), Graphical software for visualization of quantum chemistry computations, available at: https://www.chemcraftprog.com

[17] G. Knizia, *J. Chem. Theory Comput.*, 2013, **9**, 4834–4843.

[18] G. Knizia and J. E. M. N. Klein, *Angew. Chem., Int. Ed.*, 2015, **54**, 5518–5522.
